# A Helminth Immunomodulator Exploits Host Signaling Events to Regulate Cytokine Production in Macrophages

**DOI:** 10.1371/journal.ppat.1001248

**Published:** 2011-01-06

**Authors:** Christian Klotz, Thomas Ziegler, Ana Sofia Figueiredo, Sebastian Rausch, Matthew R. Hepworth, Nadja Obsivac, Christine Sers, Roland Lang, Peter Hammerstein, Richard Lucius, Susanne Hartmann

**Affiliations:** 1 Department of Molecular Parasitology, Humboldt-University Berlin, Berlin, Germany; 2 Institute for Theoretical Biology, Humboldt-University Berlin, Berlin, Germany; 3 Institute of Pathology, Charite Universtitätsmedizin Berlin, Berlin, Germany; 4 Institute of Clinical Microbiology, Immunology and Hygiene, University Hospital Erlangen, Erlangen, Germany; NIAID/NIH, United States of America

## Abstract

Parasitic worms alter their host's immune system to diminish the inflammatory responses directed against them, using very efficient immunomodulating molecules. We have previously shown that the helminth immunomodulator cystatin (AvCystatin) profoundly reduces the progression of inflammatory diseases via modulation of macrophages. Here we elucidate the signaling events in macrophages triggered by AvCystatin. Labeled AvCystatin was predominantly taken up by macrophages and subsequently induced the phosphorylation of the mitogen-activated protein kinases (MAPK) ERK1/2 and p38. IL-10 expression induced by AvCystatin in macrophages was tyrosine kinase sensitive and dependent on activation of both MAP kinases, in clear contrast to expression of IL-12/23p40. In addition, phosphorylation of the transcription factors CREB and STAT3 was induced by AvCystatin and regulated by phospho-ERK. Chemical inhibition of phosphoinositide 3-kinase (PI3K) reduced AvCystatin-induced cytokine release; however, AKT, the downstream target of PI3K, was not activated following AvCystatin exposure. To characterize signaling elements involved in alteration of the macrophage phenotype we applied mathematical modeling. Experimental testing of the *in silico* generated hypotheses identified dual specificity phosphatase (DUSP) 1 and 2, as regulators in AvCystatin triggered macrophages *in vitro* and *in vivo*. In particular, DUSP1 was subsequently found to be responsible for regulation of ERK- and p38-phosphorylation and controlled the IL-10 expression in macrophages by AvCystatin. Thus, we show that AvCystatin exploits activation and deactivation pathways of MAP kinases to induce regulatory macrophages. This study provides insights into molecular mechanisms of macrophage manipulation by parasites and highlights the utility of mathematical modeling for the elucidation of regulatory circuits of immune cells.

## Author Summary

Helminths have the ability to interfere with their host's immune response, thus downregulating inflammatory responses. We previously demonstrated the role of helminth infections or isolated helminth proteins in suppressing bystander immune responses in mouse models of allergy and colitis via a macrophage and IL-10 dependent mechanism. The current study elucidates the signaling events induced by the parasite immunomodulator AvCystatin, leading to alteration of the macrophage phenotype. AvCystatin was predominantly taken up by macrophages and induced cytokine production by phosphorylation of mitogen-activated protein kinases (MAPK) ERK1/2 and p38. To identify molecules involved in the regulation of IL-10 production we developed a mathematical model. *In silico* generated data suggested a negative feedback mechanism via deactivating ERK1/2 and p38. Ensuing experiments validated the model and revealed AvCystatin-induced dual specificity phosphatases (DUSPs) as negative regulators of MAPK activation and IL-10 expression *in vitro* and *in vivo*. Taken together, the nematode immunomodulator AvCystatin targets activating and deactivating pathways of MAPK to induce regulatory macrophages.

## Introduction

Parasitic worms have developed intricate strategies to downregulate inflammatory host immune responses directed against intruding pathogens [1], [2]. Such mechanisms of regulation are thought to establish a balanced immunological state in the host, often termed as a “modified T helper type 2 (Th2) response” that facilitates a long reproductive phase of the parasites. As an additional effect, inflammatory responses directed against unrelated antigens may also be inhibited by helminth infections (see [3], [4], [5], [6] for review). The clinical relevance of nematode-induced immunomodulation was demonstrated by the successful treatment of humans suffering from inflammatory bowel diseases with eggs from the intestinal pig whip worm *Trichuris suis* [7], [8]. The disease-alleviating effect of living parasites could also be replicated by applying single nematode proteins in mouse models of allergic and inflammatory diseases [9], [10], [11]. Thus, it is of great interest to dissect the molecular mechanisms that enable such helminth immunomodulators to limit inflammatory responses, with the goal to eventually exploit those pathways for the management of undesired immune responses.

Cysteine protease inhibitors (cystatins) of tissue dwelling filarial nematodes have been shown to be strong immunomodulators [12], [13]. These proteins are produced and secreted by all stages of the parasites. Our previous studies with recombinant cystatins from the human pathogenic filaria *Onchocerca volvulus* and the rodent filaria *Acanthocheilonema viteae* revealed an efficient induction of pro- and anti-inflammatory responses characterized by production of tumor necrosis factor (TNF)-α and high levels of interleukin (IL)-10, which eventually gave rise to an overall regulatory milieu [14], [15], [16]. Another feature of filarial cystatins is the potent interference with antigen processing and presentation by inhibiting host proteases, as shown for cystatin (CPI-2) from *Brugia malayi* [17], [18]. The main target cell of cystatin *in vitro* and *in vivo* are monocytes/macrophages [14], [15], [16]. Application of AvCystatin (Av17) strongly reduced allergic airway hyperreactivity in mouse models for asthma and colitis, respectively [11]. This effect was mediated by macrophages and dependent on IL-10. Hence, we hypothesized that filarial cystatin induces a regulatory type of macrophage characterized by the production of IL-10.

The general function of IL-10 is to limit and suppress immune responses by interfering with various functions, such as antigen presentation of dendritic cells (DC) and macrophages [19], [20]. IL-10 is therefore a potent regulator of various types of T cell responses indicated by the potential to inhibit Th1 based cytokine responses such as interferon (IFN)-γ, but also Th2 responses characterized by IL-4 and IL-13 production. IL-10 contributes significantly to the regulatory milieu necessary for the control of tissue damage and immune pathology. It is also a major player in regulating inflammatory diseases such as allergies and autoimmune disorders [20], [21]. Macrophages are both a major source and target of IL-10 [22].

A specific type of regulatory macrophage (Type 2 or M2b) has been characterized by hypersecretion of IL-10 upon stimulation of Fc-γ receptors in combination with other pro-inflammatory stimuli. The production of IL-10 by these macrophages is critically dependent on two mitogen-activated protein kinases (MAPK), extracellular signal-regulated kinase (ERK) and p38 [23], [24], [25]. Further studies showed an additional role for the phosphoinositide-3-kinase (PI3K)/AKT pathway in conversely regulating the production of IL-10 and pro-inflammatory cytokines such as IL-12 [26]. The response of macrophages to extracellular stimuli such as cytokines or TLR ligands is limited by induction of negative regulators like dual specificity phosphatases (DUSPs) or suppressor of cytokine signaling (SOCS), which act on different steps in the signaling cascade [27], [28]. The expression of suppressive factors like IL-10 is transient but may lead to prolonged effects mediated by the same phenotypically altered cell. The temporal nature of IL-10 expression in macrophages is also reflected by transient epigenetic modifications at the IL-10 promoter site, which together with the regulation of other genes may lead to different types of regulatory macrophages [19], [29].

Our previous observation of IL-10 induction in primary macrophages by filarial AvCystatin, prompted us to develop a mathematical model on IL-10 regulation, which suggested an involvement of ERK and p38 in the regulation of IL-10 production of macrophages after AvCystatin stimulation [30]. The present paper describes experimental proof of MAPK pathways as target and reports on theoretical indications and subsequent experimental validation of a feedback mechanism regulating the IL-10 production of AvCystatin-induced macrophages. We further provide evidence that the pathways addressed by AvCystatin also regulate IL-12 production in macrophages.

Hence, we characterize key molecular mechanisms of macrophage modulation by a product of parasitic worms that has potential to downregulate concomitant undesired inflammatory responses.

## Results

### Macrophages are the primary target cells of AvCystatin within cells of the peritoneal cavity

**Figure 1:**
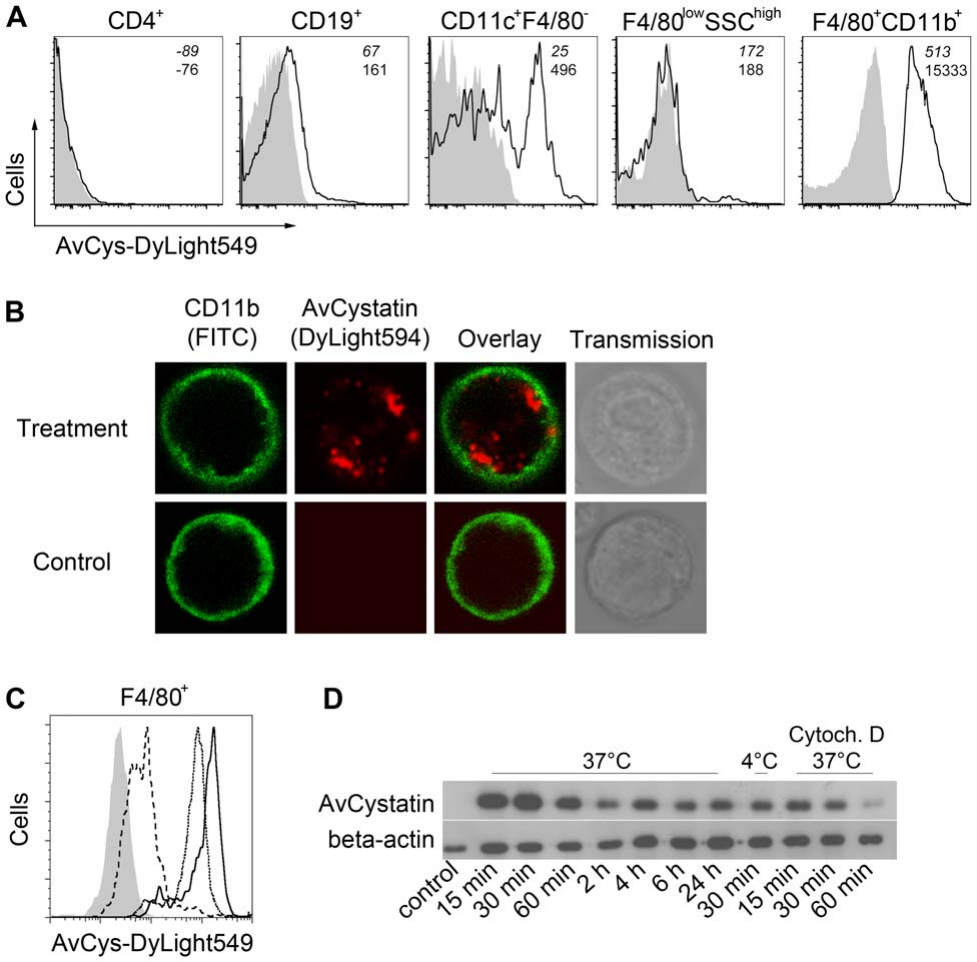
Uptake of AvCystatin by peritoneal macrophages. (A) DyLight549 labeled AvCystatin was injected into the peritoneum of BALB/c mice (40 µg/mouse) and PEC were isolated 20 min after treatment, immunostained with antibodies against CD4, CD19, CD11c, CD11b and F4/80 and analyzed by flow cytometry. Cells from untreated animals are shown in grey. (B) DyLight594 labeled AvCystatin (red) was injected into the peritoneum of BALB/c mice (40 µg/mouse) and PEC were isolated 20 min after treatment, immunostained with anti-CD11b (green) and life cells were analyzed by confocal microscopy. (C) PEC from BALB/c mice were incubated with 5 µg/ml DyLight549 labeled AvCystatin *in vitro* for 30 min at 37°C (solid line) or 4°C (long dashes). Some samples were treated with 10 µM cytochalasin D along with labeled AvCystatin at 37°C (dotted line). Cells were stained with anti-F4/80 and analyzed for DyLight549 signals by flow cytometry. Control cells without AvCystatin are shown in grey. (D) Peritoneal macrophages from BALB/c mice were incubated with 0.5 µM AvCystatin at 37°C or 4°C. To block active uptake by endocytosis some samples were pre-treated with 10 µM cytochalasin D (Cytoch. D). After indicated times excessive AvCystatin was removed by extensive washing and protein extracts were analyzed by western blot with antibodies against AvCystatin and beta-actin. (A–D) Representative experiments of 2–3 are shown.

In previous studies we demonstrated downregulation of inflammatory responses in a mouse model of asthma by applying AvCystatin into the peritoneal cavity [11]. To elucidate the primary target cells of AvCystatin in the peritoneum we analyzed the fate of fluorescence labeled AvCystatin in peritoneal exudate cells (PEC). PEC were isolated 20 min after injection of labeled AvCystatin into the peritoneum of BALB/c mice, immunostained for various cell surface markers and analyzed by flow cytometry. AvCystatin was detectable in mononuclear phagocytes such as macrophages (F4/80^+^CD11b^+^) and dendritic cells (DC) (CD11c^+^F4/80^-^) (Fig. 1A). The described macrophage and DC populations accounted for about 35% and 1.5% of the total PEC, respectively, suggesting that resident macrophages are the predominant target cells for AvCystatin within the PEC population. We did not detect any AvCystatin labeled Gr1^high^ cells in our PEC samples, revealing that AvCystatin was not internalized by infiltrating granulocytes or Gr1^high^ monocytes (data not shown). No significant binding or uptake was detectable in CD19^+^ B cells, CD4^+^ T cells or granulocytic F4/80^low^SSC^high^ cells, excluding these cells as primary targets for AvCystatin (Fig. 1A). The *in vivo* uptake of AvCystatin by CD11b^+^ phagocytes was also visualized by confocal microscopy. The punctuate signal of labeled AvCystatin suggested an uptake into the endo-lysosomal compartment (Fig. 1B).

To distinguish between specific binding and active uptake of the protein, respectively, freshly isolated PEC were incubated *in vitro* with labeled AvCystatin for 30 min at 4°C or 37°C and F4/80^+^ cells (mainly macrophages) were analyzed by flow cytometry. AvCystatin was clearly detectable in F4/80^+^ cells at 37°C, but hardly at 4°C (Fig. 1C). This suggested an active endocytotic uptake of the protein by F4/80^+^ macrophages, but little or no binding to the cell surface. Co-incubation of PEC with Cytochalasin D, a drug that inhibits endocytosis by blocking actin polymerization, revealed reduced AvCystatin uptake (Fig. 1C). Thus, macrophages are the primary target cells within the PEC population and take up AvCystatin by endocytosis.

To determine the kinetic of AvCystatin uptake in macrophage cultures western blotting was performed during a time course of incubation with AvCystatin. These experiments revealed a rapid and prolonged uptake of AvCystatin into macrophages between 0h and 24 h at 37°C (Fig. 1D). Incubation at 4°C for 30 min or at 37°C along with Cytochalasin D for 15–60 min revealed drastically reduced protein uptake supporting the flow cytometric results (Fig. 1D).

### IL-10 and IL-12/23p40 production in macrophages induced by AvCystatin are regulated by the MAPK ERK1/2 and p38 but not by Jun N-terminal kinase (JNK)

**Figure 2:**
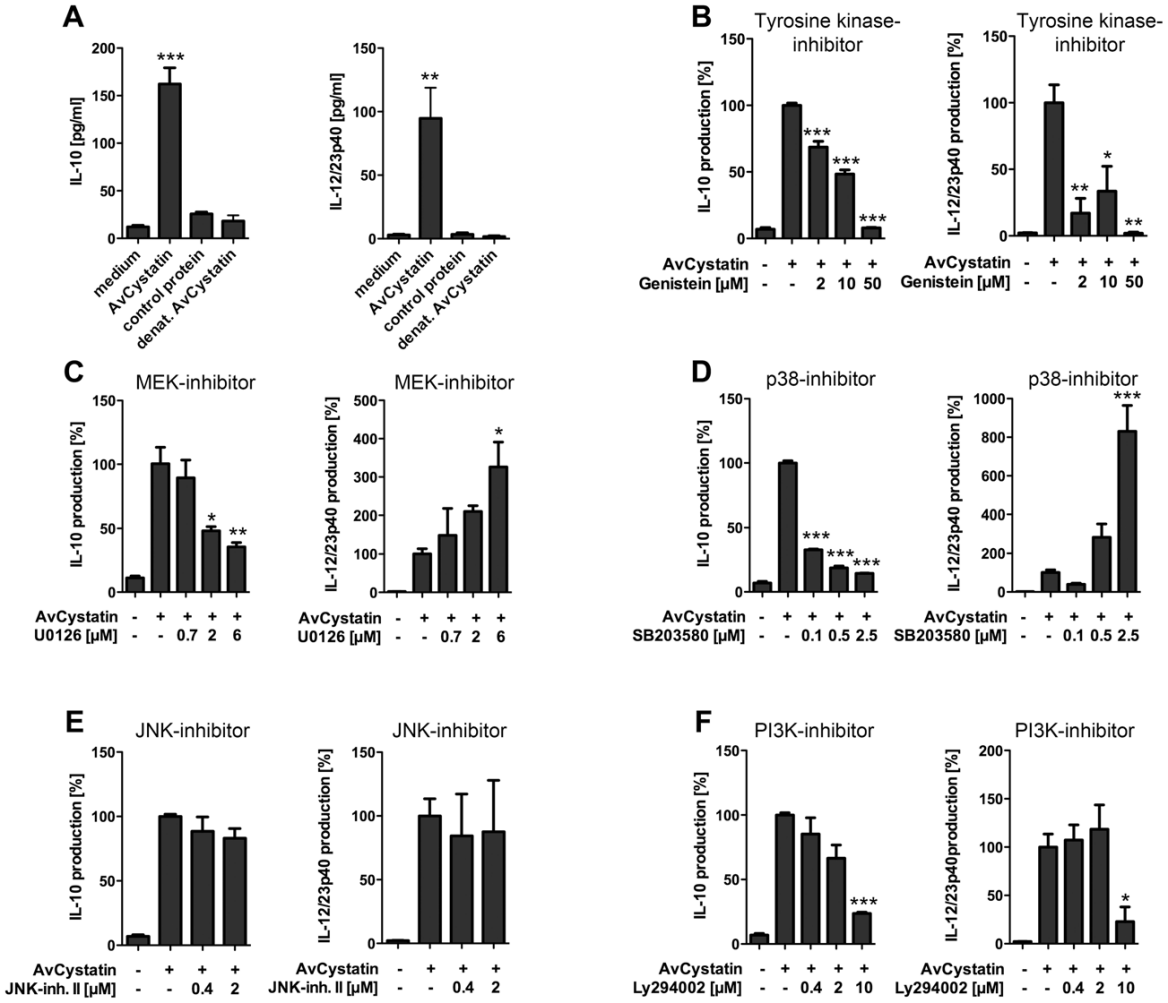
AvCystatin induced IL-10 and IL-12/23p40 production in mouse macrophages is differentially dependent on ERK, p38 and PI3K signaling. (A) Peritoneal macrophages from BALB/c mice were stimulated for 18 h with 0.5 µM AvCystatin, the same amount of denaturated AvCystatin or a control protein. (B–F) Stimulation of macrophages with 0.5 µM AvCystatin for 18 h in the presence of various concentrations of Genistein (B, broad range tyrosine kinase inhibitor), U0126 (C, MEK1/2 inhibitor), SB203580 (D, p38-inhibitor), JNK inhibitor II (E) or LY294002 (F, PI3K-inhibitor). (A–F) IL-10 and IL-12/23p40 production was determined in cell supernatants by ELISA. Data including inhibitor tests are presented as percent cytokine production compared to AvCystatin treatment. Representative experiments of 2-4 for each analysis are shown. p<0.05 (*), p<0.01 (**), p<0.001 (***).

Previous studies indicated that filarial cystatins induce pro- and anti-inflammatory cytokines in mononuclear phagocytes and give rise to an overall regulatory cell type able to abrogate inflammatory effects in a mouse model of asthma [11], [16], [31]. Here, we studied the signaling events induced by AvCystatin leading to IL-10 and IL-12/23p40 expression in primary peritoneal macrophages. We analyzed the cytokine production in cell supernatants of AvCystatin-stimulated macrophages in the presence of various inhibitors of molecules known to be involved in regulation of IL-10 and IL-12/23p40 expression (Fig. 2). First, we confirmed the significant induction of IL-10 and IL-12/23p40 in macrophages after AvCystatin stimulation (Fig. 2A). Next, we applied the broad range tyrosine kinase inhibitor Genistein and found a dose dependent reduction of IL-10 and IL-12/23p40 production after AvCystatin stimulation, indicating the involvement of a tyrosine kinase sensitive signaling leading to cytokine production (Fig. 2B).

Further, we co-incubated cells with the immunomodulator and a MEK1/2 specific inhibitor (U0126, IC_50_  = 72 and 58 nM, respectively) that blocks ERK1/2 phosphorylation. Likewise, we used a p38 MAPK specific inhibitor (SB203580, IC_50_ = 0.3–0.5 µM). Both inhibitors exerted a strong and concentration dependent reduction of AvCystatin-induced IL-10 production (Fig. 2C, D). In contrast, inhibition of p38 and MEK1/2 increased the production of IL-12/23p40 in a dose dependent manner (Fig. 2C, D). The inhibition of the MAPK JNK by application of up to 2 µM JNK-inhibitor II (IC_50_ = 40–90 nM) had no significant effect on the AvCystatin-induced IL-10 and IL-12/23p40 production by macrophages (Fig. 2E). In conclusion, these data indicated that AvCystatin addresses the ERK- and p38-pathway to induce IL-10 and to regulate IL-12/23p40 production.

The PI3K signaling pathway is important for IL-10 and IL-12 regulation in TLR stimulated macrophages [26]. To investigate a possible regulatory role of the PI3K–AKT signaling pathway in AvCystatin-induced cytokine production we tested the effect of a specific PI3K inhibitor, LY294002 (IC_50_  = 1.4 µM). Both, IL-10 and IL-12/23p40 production were inhibited by an inhibitor concentration of 10 µM (Fig. 2D).

Hence, these results clearly suggest the involvement of a tyrosine kinase sensitive and ERK/p38 dependent mechanism for AvCystatin-induced IL-10 production in macrophages. In addition, the activation of these pathways contributes to the regulation of IL-12/23p40 production by macrophages.

### AvCystatin induces transient activation of ERK1/2 and p38

**Figure 3:**
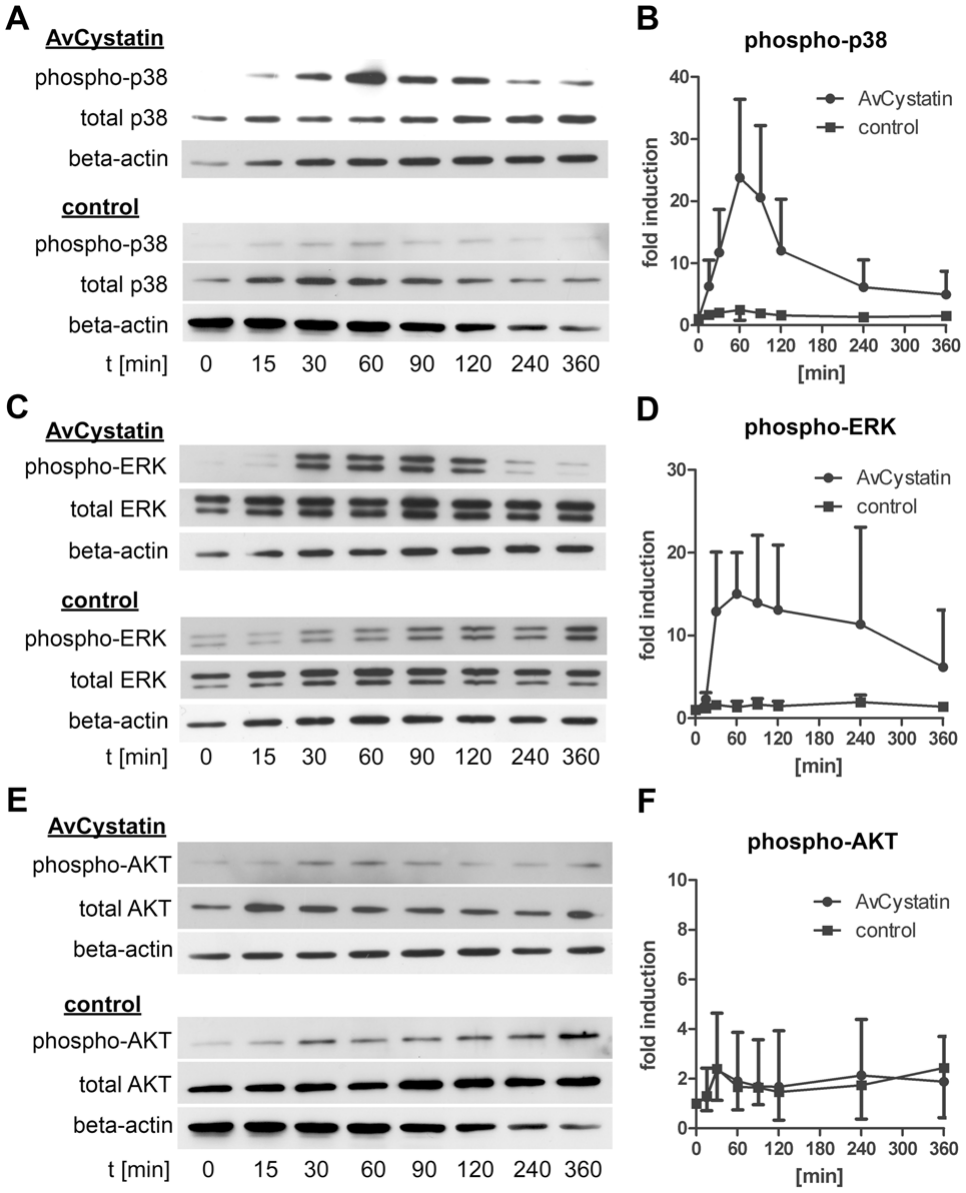
AvCystatin stimulation induces transient phosphorylation of p38 and ERK. Thioglycollate elicited peritoneal macrophages from BALB/c mice were stimulated with 0,5 µM AvCystatin or the same amount of denaturated AvCystatin as a control. After the indicated time points total cell extracts were isolated and analyzed by western blot with antibodies against phospho-p38, total-p38, phospho-ERK, total-ERK, phospho-AKT, total-AKT and beta-actin. (A, C, E) Representative blots are shown. (B, D, F) Densitometric analysis (mean ± SD, n = 3–4) of phospho-p38, phospho-ERK and phospho-AKT normalized to the endogenous control are expressed as fold induction relative to the medium control.

As MAPK were shown to be essential for IL-10 production and regulation of IL-12/23p40 we analyzed the kinetics of ERK1/2 and p38 activation. Macrophages were incubated with AvCystatin or under control conditions and cell lysates of various time points were analyzed by western blotting. The first AvCystatin-induced p38 phosphorylation was seen after 15 min, increased to a maximum level at 60 min and then dropped between 60–120 min to almost background levels at 360 min (Fig. 3A, B). ERK1/2 phosphorylation reached its maximum at 60 min after stimulation and remained high between 30–120 min; the signal finally decreased between 120–360 min (Fig. 3C, D).

Next, we tested whether AvCystatin leads to activation of AKT. Surprisingly, although the inhibitor LY294002 indicated a role for PI3K pathway in AvCystatin induced cytokine production no specific phosphorylation of AKT was detectable after AvCystatin stimulation (Fig. 3E, F). These results indicate that AvCystatin does not induce phosphorylation of AKT. This suggests that endogenous, physiological levels of phospho-AKT suffice for optimal cytokine production in macrophages after AvCystatin stimulation.

We conclude that AvCystatin induces a transient activation of p38 and ERK leading to IL-10 production and regulation of IL-12/23p40 expression.

### AvCystatin-stimulated signaling pathways ERK1/2 and p38 are partially linked

Cross regulation and hierarchical phosphorylation steps are important in intracellular signaling processes. To determine possible cross-talk between the involved kinases in AvCystatin-stimulated macrophages and to define a potential hierarchical signaling cascade between p38, ERK and AKT in our system we compared the phosphorylation signals of p38, ERK and AKT after stimulation with AvCystatin in the presence of specific inhibitors and after various times.

**Figure 4:**
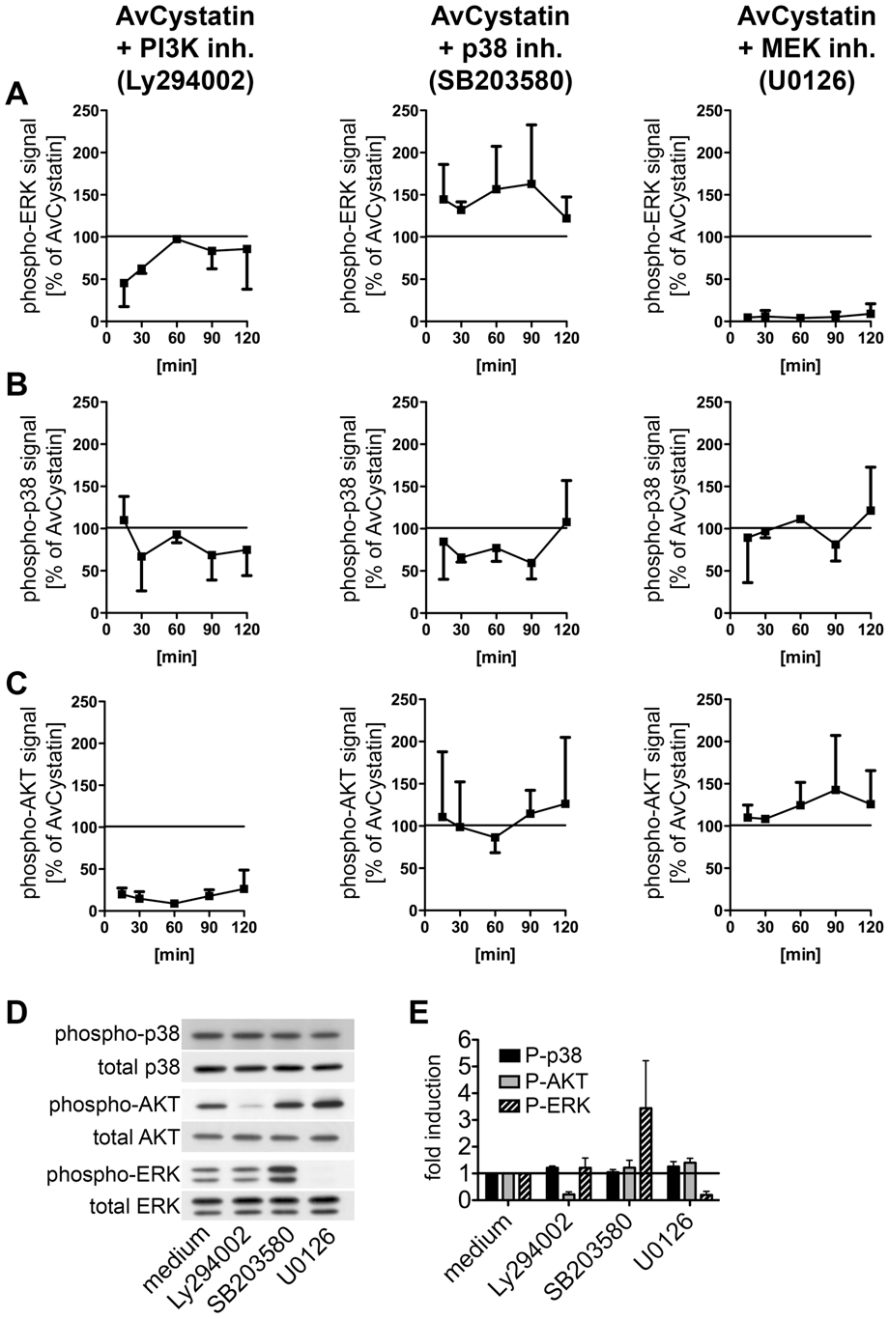
AvCystatin stimulated signaling pathways are partially connected but are independently addressed by AvCystatin. (A–C) Thioglycollate elicited peritoneal macrophages from BALB/c mice were treated with 0.5 µM AvCystatin in the presence of LY294002 (5 µM, PI3K inhibitor), SB203580 (0.5 µM, p38 inhibitor) or U0129 (5 µM, MEK1/2 inhibitor). After indicated times total cell extracts were isolated and analyzed by western blot using antibodies against phospho-p38, phospho-ERK, phospho-AKT and respective total proteins. See figure S1 for examples of original western blots. Densitometric analysis of (A) phospho-ERK, (B) phospho-p38 and (C) phospho-AKT is presented as mean percentage (median ± range, n = 2) normalized to the AvCystatin positive control of each time point ( = 100%). (D, E) In control experiments non-stimulated cells were treated with inhibitors, as described above, for 60 min and cell extracts were analyzed by western blot of phospho-proteins and their respective controls. (D) Representative blots are shown. (E) Densitometric analysis (median ± range, n = 2) of phospho-proteins normalized to the respective total protein is expressed as fold induction relative to the medium control.

Western blot analysis of phospho-ERK1/2 revealed that inhibition of PI3-kinase led to a transient reduction by 50% of ERK phosphorylation at 15–30 min after AvCystatin treatment (Fig. 4A, Fig. S1). In contrast, inhibition of p38 during AvCystatin treatment resulted in approximately 50% stronger ERK1/2 phosphorylation signals compared to the ERK1/2 stimulation by AvCystatin only, indicating a regulatory effect of p38 on ERK phosphorylation (Fig. 4A, Fig. S1). The ERK-inhibitor showed a drastic inhibition of phospho-ERK, confirming its activity.

Induction of phospho-p38 by AvCystatin was not influenced by inhibition of ERK, whereas inhibition of PI3K had a slight, but not significant effect (Fig. 4B, Fig. S1). Hence, AvCystatin-induced phospho-p38 is independent of active forms of ERK and AKT. It should be noted that the p38-inhibitor SB203580 directly inhibits the kinase activity of p38, but does not prevent its phosphorylation. Consequently, we still detected phospho-p38 signal in the presence of SB203580, although the inhibitor was functional (Fig. 4B, Fig. S1).

Phospho-AKT signal was neither altered by the inhibition of p38 nor by the inhibition of ERK1/2 (Fig. 4C, Fig. S1), whereas inhibition of PI3K drastically reduced the phospho-AKT signal confirming the activity of the inhibitor (Fig. 4C, Fig. S1).

Control experiments with inhibitors revealed that the treatment of non-stimulated cells with the p38 inhibitor led to a strong increase of phospho-ERK1/2 (Fig. 4D, E). This increase was comparable to the effect of the inhibitor in AvCystatin treated cells (Fig. 4A, Fig. S1). The inhibitor had no effect on phosphorylation of AKT. Treatment of non-stimulated cells with the inhibitors for p38 and AKT had no influence on phosphorylation levels of p38 or AKT and p38 or ERK, respectively.

Taken together, these data indicate that AvCystatin activates ERK1/2 and p38 independently and that phospho-p38 inhibits the strength of ERK1/2 phosphorylation. In addition, early activation of ERK1/2 (between 0–30 min) is partly dependent on endogenous levels of phospho-AKT, which might explain the above-mentioned IL-10 reduction after inhibition of PI3K-AKT.

### Activation of transcription factors by AvCystatin

**Figure 5:**
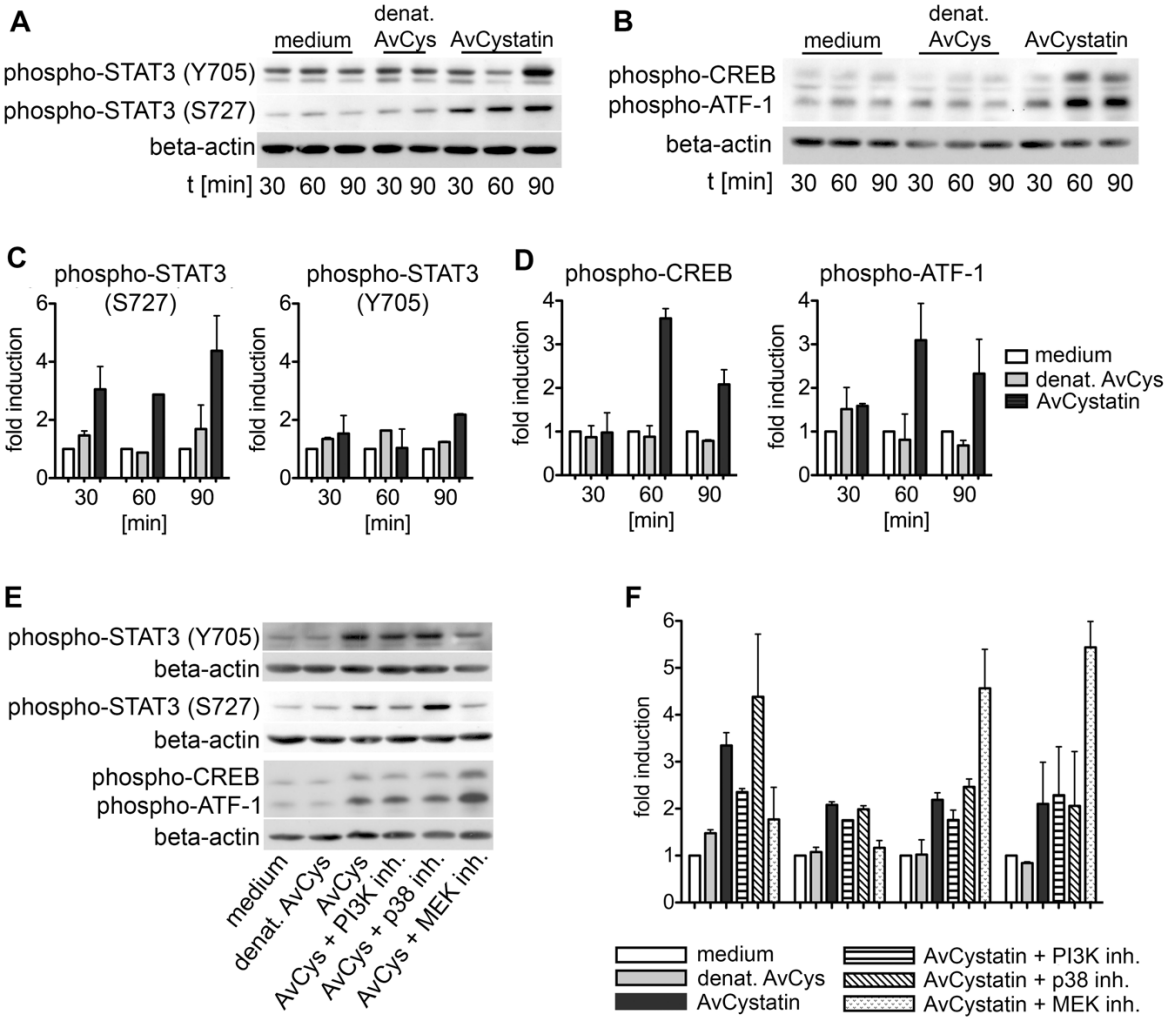
AvCystatin induces phosphorylation of transcription factors CREB and STAT3. (A, B) Thioglycollate elicited peritoneal macrophages from BALB/c mice were treated with 0.5 µM AvCystatin or the same volume of denaturated AvCystatin as a control. After indicated times total cell extracts were isolated and applied in western blot analysis using antibodies against phospho-CREB/ATF-1 and phospho-STAT3 (Y705) or phospho-STAT3 (S727), respectively, and beta-actin. Exemplary blots are shown. (C, D) Densitometric analysis of phosphorylation signals of (A) and (B) were normalized to beta-actin and are presented as fold induction (median ± range, n = 2) relative to the appropriate medium control. (E) Stimulation of macrophages with 0,5 µM AvCystatin in the presence of various inhibitors, MEK1/2 inhibitor (U0126, 5 µM), p38-inhibitor (SB203580, 0,5 µM) and PI3K-inhibitor (LY294002, 5 µM). Cells were stimulated for 90 min and western blot analysis were performed as described in (A) and (B). Exemplary blots are shown. (F) Densitometric analysis of phosphorylation signals of (E) were normalized to beta-actin and are presented as fold induction (median ± range, n = 2) relative to the appropriate medium control.

To further define the intracellular events induced by AvCystatin in macrophages we analyzed the activation of two important transcription factors for the regulation of IL-10 and IL-12/p40 expression, signal transducer and activator of transcription 3 (STAT3) and cAMP response element-binding protein (CREB)/activating transcription factor 1 (ATF1), in cell lysates generated 30–90 min after AvCystatin stimulation. Phosphorylation of STAT3 and CREB can be directly or indirectly mediated by both MAPK p38 and ERK1/2 [32], [33]. AvCystatin induced the phosphorylation of both STAT3 and CREB/ATF1 (Fig. 5). Phosphorylation of STAT3 at Serine 727 was already 3 fold higher after 30 min and Tyrosine phosphorylation was 2 fold higher 90 min after AvCystatin stimulation (Fig. 5A, C). Similarly, CREB/ATF1 was activated at 60–90 min after stimulation (Fig. 5B, D).

Furthermore, we tested whether the inhibition of p38, ERK and PI3K had a direct effect on transcription factor phosphorylation in our system. Inhibition of MEK/ERK with U0126 totally blocked the phosphorylation of STAT3 (Tyr705 and Ser727) but increased the phosphorylation of CREB/ATF1 (Fig. 5E, F). Surprisingly, inhibition of p38 with SB203580 had no effect on STAT3 and CREB/ATF1, and inhibition of PI3K with LY294002 slightly inhibited the phosphorylation of STAT3 (S272) but not of STAT3 (Y705) or CREB/ATF1.

Taken together, these data show that AvCystatin-induced MAPK signaling differentially mediated the activation of the transcription factors STAT3 and CREB/ATF1, which are both commonly involved in regulation of cytokine transcription.

### Modeling of AvCystatin-induced IL-10 expression in macrophages shows regulation by DUSPs

To further identify the signaling mechanisms leading to the regulatory macrophage phenotype determined by AvCystatin we focused on the IL-10 expression and regulation. One efficient way to test various hypotheses based on known literature is mathematical modeling. Via mathematical modeling we were able to first undertake *in silico* analyses prior to performing experiments. Once the model has been implemented, additional *in silico* sensitivity analysis helped to test how the system behaves when specific parameters are changed. Such questions are difficult to tackle and mathematical modeling helped to describe specific characteristics. In the present work, the understanding of the mechanisms of transient MAPK activation and the underlying IL-10 expression were crucial to elucidate the type of macrophage that emerges after AvCystatin treatment.

First, we adapted the mathematical model published in Figueiredo et al. 2009 in order to incorporate a negative feedback mechanism acting at the MAPK level, specifically on ERK and p38. A master model illustrates the scope of this analysis (Fig. S2). Based on this, we constructed and tested 35 specific mathematical models (Table S1) and investigated their ability to predict our experimental findings. Fig. S3 illustrates the process of model fitting, validation, verification and selection. For a detailed description of these methods please refer to the ‘material and methods’ section. Our final model was sufficient to describe the transient nature of MAPK activation and IL-10 expression.

**Figure 6:**
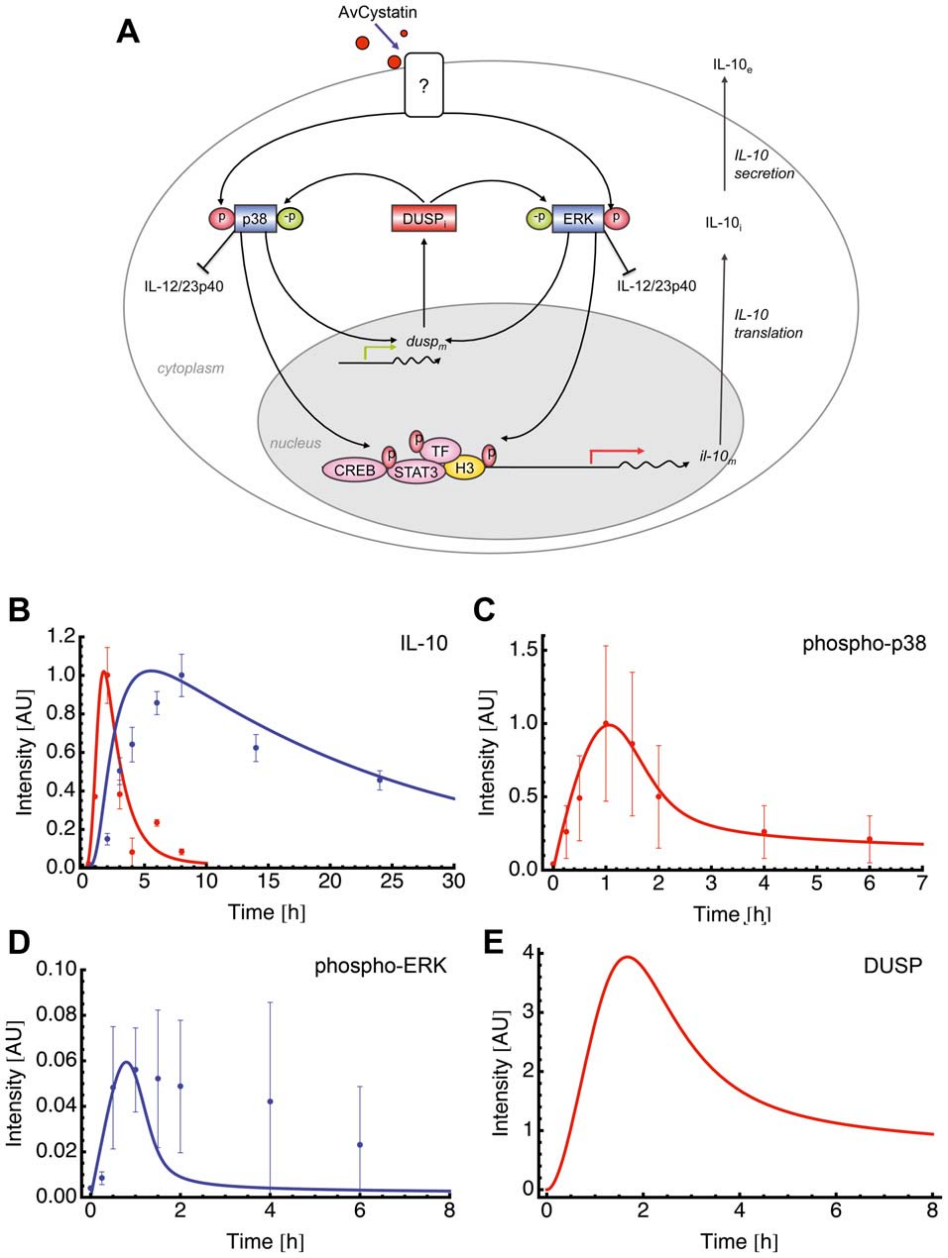
Mathematical model for AvCystatin induced IL-10 expression in macrophages. (A) Cartoon represents the principle of selected model 15 (Table S1) for induction and regulation of IL-10 based on the experimental data and theoretical considerations. AvCystatin phosphorylates p38 and ERK, both mediating downstream modulation of CREB, STAT3 and possibly other transcription factors (TF) and histone modifications (H3) of the IL-10 promoter locus. The model predicts the regulation of ERK and p38 by a dedicated phosphatase (DUSP). In addition, AvCystatin regulates the expression of IL-12/23p40 in macrophages through the activation of ERK and p38. In model 15 we only focused on induction and regulation of IL-10. (B–E) Fitting of model 15 to the data of (A) IL-10 and (B) p38, and model predictions of (C) ERK and (D) DUSP using the parameter values obtained with the model fitting patent. (B) Model fitting to IL-10 protein (blue) and IL-10 mRNA (red). Model simulations (line) are illustrated in comparison to values of experimental data (dots, mean ± SD, n = 3). (C) Model fitting to p38. Model simulation (line) is illustrated in comparison to values of experimental data (dots, mean ± SD, n = 3). (D) Model prediction for ERK (line) is illustrated in comparison to experimental data (dots, mean ± SD, n = 3). (E) Model prediction for DUSP is shown.

This specific model (model 15 of Table S1) assumed activation of p38 and ERK leading to expression of a dedicated phosphatase (such as DUSP) that in turn negatively regulates both ERK and p38 (Fig. 6A).

Fitting of the model to experimental data confirmed that this model mimics the experimental findings (Fig. 6B, C). We further compared the model predictions and experimental data for phospho-ERK and found a similar trend for ERK dynamics (Fig. 6D). The model successfully predicted a peak of ERK dynamics at time point t = 0.8 and this is supported by the experimental data. The model curve for ERK has a faster decay than experimental ERK. Additionally, the model predicted a time dependent expression of DUSP, having its maximum at time point t = 1.7 h (Fig. 6E). Taken together, the mathematical model predicts a feedback mechanism on p38 and ERK1/2 by DUSP.

### Sensitivity analysis of the mathematical model reveals autocrine crosstalk between individual components

**Figure 7:**
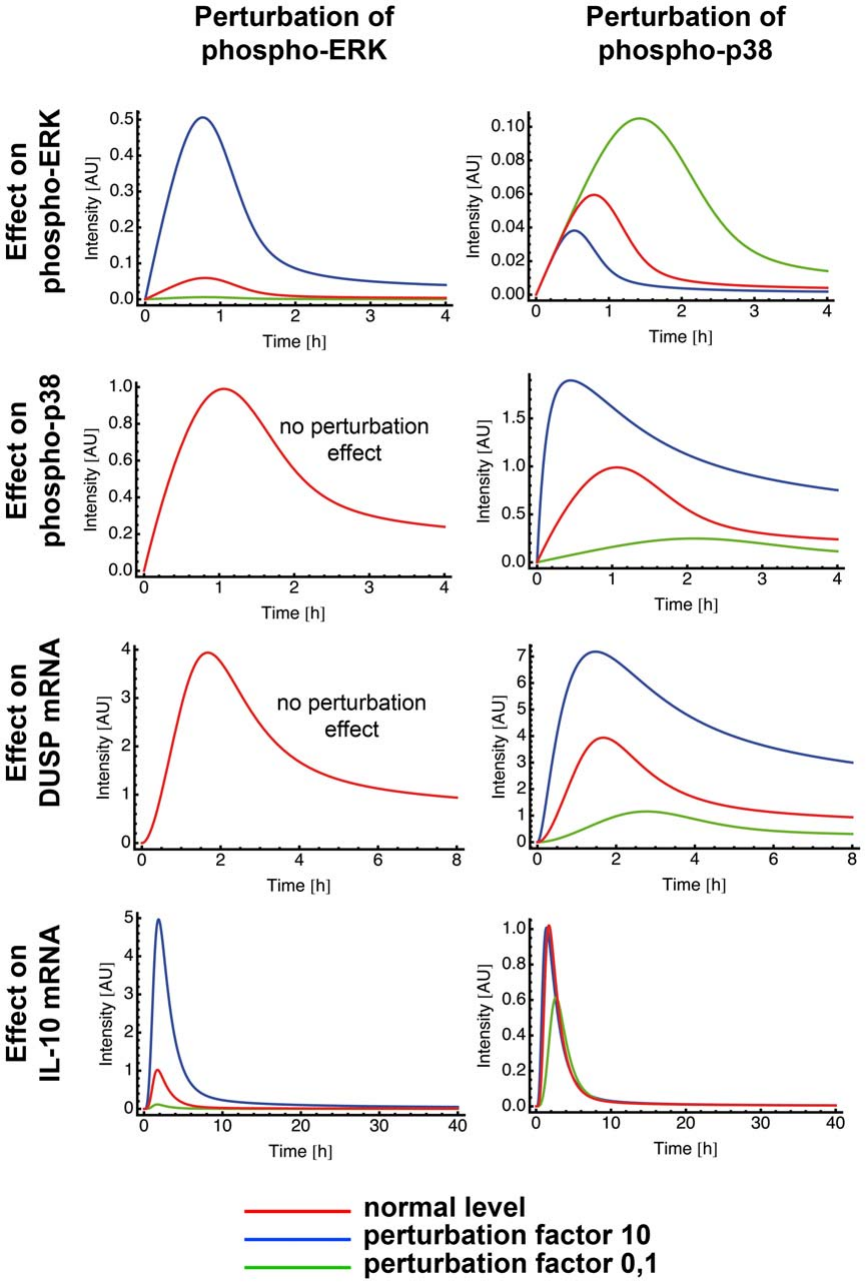
Perturbation of ERK and p38 in the mathematical model. Mathematical models can be used to make *in silico* predictions when a single parameter of a system is changed. Model 15 was used to analyze the effect of ERK and p38 perturbation on ERK, on p38, on DUSP and on IL-10 mRNA. We used the perturbation factors 10 and 0,1 to mimic a parameter increase and decrease of the indicated component, respectively.

*In silico* analysis of a biological network allows to perturb each element of the network and observe the impact of this perturbation in the other elements. We used perturbation factors of 10 (reflects increase) and 0.1 (reflects inhibition) to evaluate the effect of a higher and lower phosphorylation rate of one of the MAPK p38 and ERK, respectively, and compared them to the unperturbed values (Fig. 7).

Interestingly, perturbations in the phosphorylation rate of ERK produced no effect on p38 (Fig. 7). In contrast, higher/lower phosphorylation rate of p38 produced a decrease/increase effect on ERK phosphorylation rate (Fig. 7). The blockage of p38 in our experiments also increased the phosphorylation rate of ERK (Fig. 4) confirming the results of the model. Hence, the experimental data and the model suggested an autocrine crosstalk of p38 acting on ERK.

Perturbations of p38 phosphorylation rate produced a linear effect on DUSP expression and ERK perturbation did not affect DUSP (Fig. 7). Next, we checked whether ERK or p38 were mainly responsible for the level of IL-10 gene expression. Our model clearly showed that perturbation of the ERK phosphorylation rate had a strong and linear effect on IL-10 level. Inhibition of p38 phosphorylation levels resulted in a drastic inhibition of IL-10 expression and a total blockage of p38 completely abrogated IL-10 levels (Fig 7, and data not shown). Thus, further confirming the model by our experimental data. Amplification of p38 phosphorylation levels did not affect IL-10 expression (Fig. 7). This can be due to a possible saturation effect.

Taken together, the experimental and mathematical data suggest that ERK affects mainly IL-10 production and regulation, whereas p38 affects mainly IL-10 and DUSP activation and, indirectly, ERK regulation through an autocrine crosstalk.

### AvCystatin induces differential expression of various DUSPs

**Figure 8:**
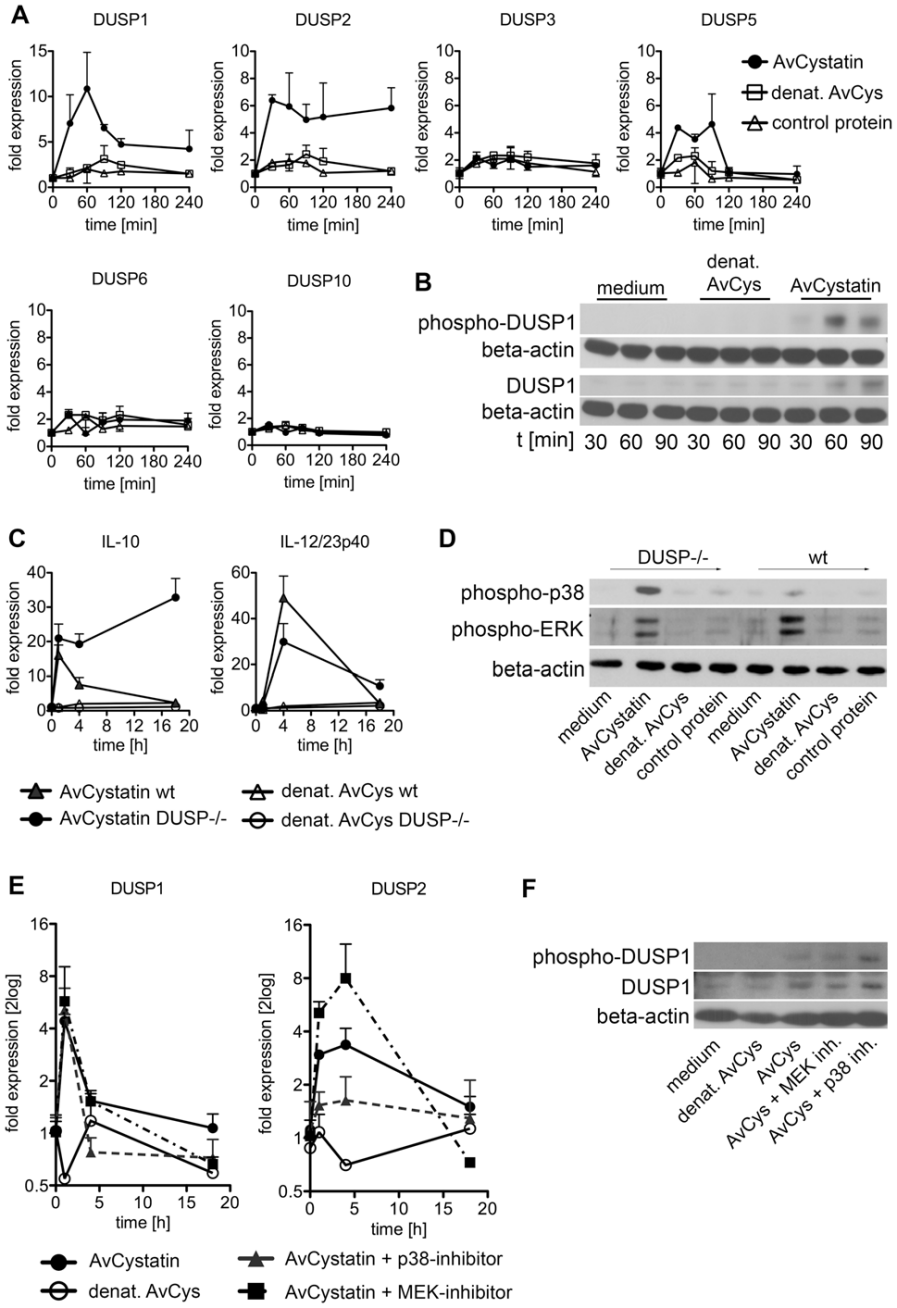
Regulated expression of DUSP1 and DUSP2 in macrophages by AvCystatin. (A, B) Thioglycollate elicited peritoneal macrophages from BALB/c mice were treated with 0.5 µM AvCystatin or the same amount of denaturated AvCystatin as a control. (A) After indicated times total RNA was extracted and real time PCR analysis was performed for DUSP1, DUSP2, DUSP3, DUSP5, DUSP6 and DUSP10. Normalized data are expressed as fold induction compared to untreated controls. Shown is one exemplary experiment done in duplicates out of 3 independent experiments, which gave similar results. (B) After indicated times total cell extracts were isolated and analyzed by western blot using antibodies against phospho-DUSP1 and DUSP1, respectively, and beta-actin. (C, D) Thioglycollate elicited peritoneal macrophages from DUSP1^-/-^ mice and wt (C57/BL6) littermates were treated with 0.5 µM AvCystatin or the same amount of denaturated AvCystatin as a control. (C) After indicated times total RNA was extracted and real time PCR analysis was performed for IL-10 and IL-12/23p40. Normalized data are expressed as fold induction compared to untreated controls. Shown is one experiment done in triplicates out of 2 independent experiments, which gave similar results. (D) After 60 min total cell extracts were isolated and analyzed by western blot using antibodies against phospho-p38, phospho-ERK and beta-actin. (E, F) Thioglycollate elicited peritoneal macrophages from BALB/c mice were treated with 0.5 µM AvCystatin in the presence of p38 inhibitor (0.5 µM, SB203580) or MEK1/2 inhibitor (5 µM, U0129). (E) After indicated times total RNA was extracted and real time PCR analysis was performed for DUSP1 and DUSP2. Normalized data are expressed as fold induction compared to untreated controls. Shown is one exemplary experiment done in triplicates out of 3 independent experiments, which gave similar results. (F) Total cell extracts were isolated 60 min after AvCystatin stimulation and analyzed by western blot using antibodies against phospho-DUSP1, DUSP1 and beta-actin.

To verify the predicted role of DUSPs, we experimentally tested the early kinetics (0–4 h) of the mRNA expression of DUSP1, DUSP2, DUSP3, DUSP5, DUSP6 and DUSP10 after AvCystatin treatment by real time PCR (Fig. 8A). These phosphatases have all been implicated in the regulation of activated MAP kinases [34]. Both DUSP1 and DUSP2 were upregulated in macrophages as early as 30 min after AvCystatin treatment. DUSP1 expression peaked at 60 min, whereas DUSP2 expression was constant between 30–240 min after AvCystatin stimulation. A slightly increased expression of DUSP5 between 30–120 min was also detected. The mRNA levels of DUSP3, DUSP6 and DUSP10 were not altered in AvCystatin treated macrophages (Fig. 8A). We mapped the DUSP expression from our experiments and model in one graph and found that the model showed a similar trend of the expression curves and a slightly delayed expression pattern compared to experimental expression of DUSP1 and DUSP2 (Fig. S4).

To substantiate the real time PCR data, we analyzed protein expression and phosphorylation of DUSP1 by western blotting (Fig. 8B). Interestingly, phosphorylation of DUSP1 can be mediated by ERK, which leads to protein stabilization [35]. DUSP1 showed increased protein expression at 60–90 min after AvCystatin stimulation confirming the data of the real time PCR and the model predictions. Furthermore, DUSP1 phosphorylation increased after 60 min and was lowered again after 90 min, indicating a stabilization of the protein (Fig. 8B).

Thus, the data correlated well with the selected mathematical model and suggested that DUSPs are relevant for AvCystatin modulation of macrophages, since they have been described as targets and regulators of p38 and ERK1/2 [27], [36], [37].

### DUSP1 regulates IL-10 expression in macrophages

To further investigate the role of DUSPs we analyzed cytokine expression and MAPK activation after AvCystatin treatment in macrophages from DUSP1^-/-^ animals. Macrophages from wild type animals showed a transient IL-10 mRNA expression after AvCystatin treatment, whereas DUSP1^-/-^ macrophages showed a sustained and increased IL-10 expression, confirming an important role of DUSP1 in IL-10 regulation in macrophages following AvCystatin treatment (Fig. 8C). IL-12/23p40 mRNA expression in wild type macrophages was transiently induced. DUSP1^-/-^ macrophages showed a slightly reduced IL-12/23p40 expression after 4 h although the overall transient expression was not altered (Fig. 8C). AvCystatin stimulated macrophages from DUSP1^-/-^ animals showed a drastic accumulation of phospho-p38 and slightly reduced phospho-ERK levels as compared to macrophages from wild type animals (Fig. 8D). In conclusion, these data are in accordance to the model predictions and highlighted the importance of DUSP1 for the regulation of MAPK activation and cytokine regulation after AvCystatin treatment.

We also assessed the converse effect of experimental MAPK inhibition on DUSP1 and DUSP2 expression. DUSP2 expression was reduced after p38-inhibition and increased after ERK-inhibition (Fig. 8E). Interestingly, DUSP1 expression was not altered by p38 and ERK-inhibition, respectively. To test whether DUSP1 is regulated on protein level we analyzed DUSP1 phosphorylation and total DUSP1 in AvCystatin-treated macrophages after p38 and ERK inhibition. The phosphorylation of DUSP1 was increased after p38 inhibition and not affected by inhibition of ERK, whereas total DUSP1 protein was slightly decreased after ERK inhibition and increased after p38 inhibition (Fig. 8F). These data indicate that in our system DUSP1 is regulated on protein level rather than on transcriptional level by ERK and p38. In light of simplicity we used only one hypothetical DUSP in our model and predicted a direct effect of p38, not ERK, on DUSP expression (Fig. 7). The discrepancies between the model and the experimental data are possibly due to the fact that various DUSPs possess differential function and therefore their behavior cannot be reflected by one representative DUSP in the model. In addition, the model does not encounter possible modifications on DUSP protein. However, the model correctly predicts an overall effect of DUSPs on MAPK regulation. Overall, these data imply that the activation of ERK and p38 in AvCystatin-stimulated macrophages differentially regulate mRNA and protein levels of DUSP1 and DUSP2 as a feedback control for the activation of ERK and p38.

**Figure 9:**
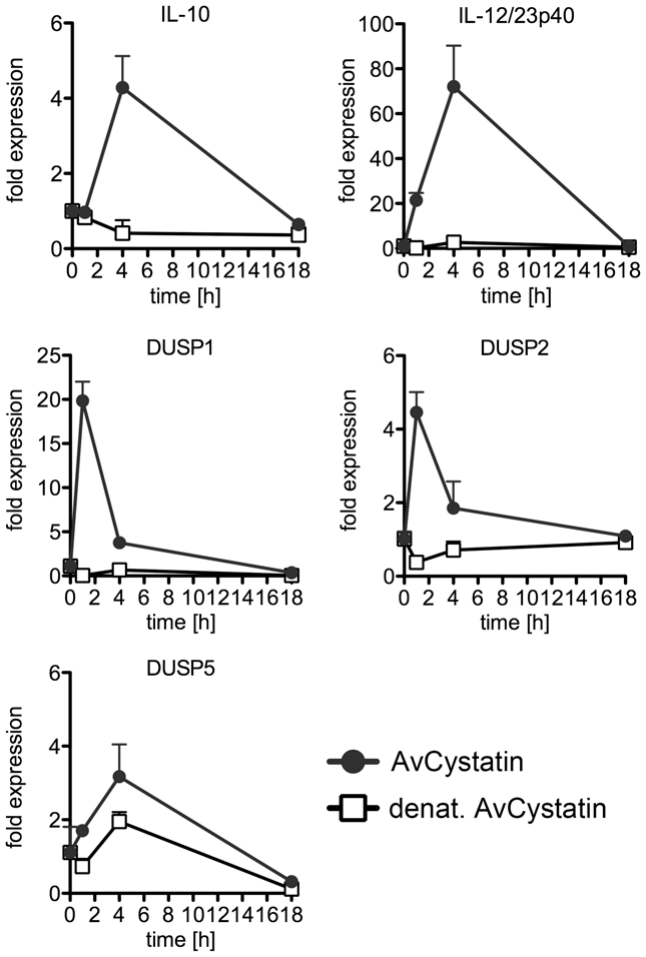
*In vivo* effect of AvCystatin confirms cytokine and DUSP expression. BALB/c mice were treated i.p. with AvCystatin (20 µg/mouse) or the same amount of denaturated AvCystatin as a control for 1 h, 4 h or 18 h. Untouched macrophages were purified from isolated PEC and applied for real time PCR analysis for IL-10, IL-12/23p40, DUSP1, DUSP2 and DUSP5. Each group consisted of pooled cells from 3-4 mice analyzed in triplicate values. The experiment was repeated twice with similar results.

Finally, to elucidate the *in vivo* relevance of our findings, we injected AvCystatin or denaturated AvCystatin as a control into the peritoneum of BALB/c mice. Peritoneal macrophages were isolated after various time points and cytokine and DUSP expression was analyzed by real-time PCR (Fig. 9). The data revealed similar transient expression patterns for IL-10 and IL-12/23p40 with a peak at 4 h after AvCystatin injection and a higher IL-12/23p40 expression. AvCystatin treatment also induced the expression of DUSP1 and DUSP2. The DUSP kinetics also reflected the *in vitro* findings with a peak at 1 h after treatment and a sharp decline after 4 and 18 h (Fig. 9).

In conclusion, AvCystatin treatment of macrophages induced the expression of DUSP1 and DUSP2 *in vitro* and *in vivo*. In particular, DUSP1 expression is responsible for the regulation of ERK- and p38-phosphorylation and controls the IL-10 expression in macrophages by AvCystatin.

## Discussion

Induction of IL-10 as an immunosuppressive cytokine is a common phenomenon found in chronic helminth infections. In the present study we elucidate the pathways leading to the induction of regulatory macrophages by applying experimental approaches and mathematical modeling. We show that the nematode immunomodulatory protein AvCystatin manipulates host macrophages by activation of the MAP kinases ERK and p38 to induce IL-10 and IL-12/23p40 production and that this activation is regulated by a feedback mechanism involving DUSPs. These data provide underlying molecular mechanisms for our previous report on the ameliorating effect of AvCystatin on inflammatory immune responses [11].

Our results support findings on regulatory type 2 macrophages (M2b) that are characterized predominantly by high IL-10 production. M2b macrophages can be generated after activation of p38 and ERK1/2 by crosslinking of Fc-γ receptors plus inflammatory signals such as TNF-α [38], [39], [40]. Detailed analyses revealed that activation of ERK1/2 leads to a fast and transient phosphorylation of histone H3 specifically at the IL-10 promoter. This transient modification renders the IL-10 promoter accessible for binding of transcription factors such as Sp1 and STAT3 that are provided by the parallel activation of p38 [23], [25]. Additionally, activation of CREB leads to promoter binding and positively correlates with IL-10 production in macrophages [26]. Interestingly, in macrophages unlike in other cell types transient phosphorylation of histones but not acetylation of the IL-10 promoter site correlates with the dynamics and control of the IL-10 gene expression [19], [23]. Furthermore, inhibition studies suggested that histone phosphorylation is mainly ERK1/2 and partly p38 dependent. Further studies have to show whether transient histone phosphorylation is the mode of promoter activation resulting from stimulation with AvCystatin.

M2b Macrophages need two pro-inflammatory stimuli to become regulatory as characterized by high IL-10 production, as well as high levels of iNOS expression leading to NO production [41]. In this respect, AvCystatin generated regulatory macrophages show remarkable similarities to M2b macrophages. Both cell types induce IL-10 mediated immune regulation *in vivo* and both the M2b macrophages and AvCystatin-induced macrophages, additionally show a strong increase of the classical activation markers such as iNOS *in vitro* [13], [39]. Due to these profound similarities we hypothesize that AvCystatin might also address cooperating receptors for macrophage modulation. So far, no specific receptor for endogenous or parasite cystatins on macrophages or other immune cells has been identified. Therefore, it remains open whether the described AvCystatin effects are due to receptor engagement or to inhibitory effects on cysteine proteases of target cells or a combination of both mechanisms.

Our data on binding and uptake support the findings of Ekström et al. (2008) on active internalization of human cystatin C into capan-1 tumor cells. The internalization was blocked by excess of unlabeled cystatin C indicating specific uptake [42]. Human cystatin C has been shown to inhibit epithelial-mesenchymal transition of mammary tumor cells via antagonized binding to the TGF-β receptors on these tumor cells [43], [44]. We tested whether AvCystatin also interferes with TGF-β signaling by using a TGF-β reporter cell system [45], but found no evidence for direct activation or inhibition of TGF-β receptor mediated signaling, thus excluding TGF-β receptor as target of AvCystatin on macrophages (unpublished data). Alternatively, AvCystatin might directly or indirectly address pattern recognition receptors. In general, the recognition of helminth molecules by innate immune cells is not well defined yet and probably no common receptor for the recognition of helminths and their products exist [46]. However, a few defined molecules have been identified and shown to interact with toll like receptors (TLR) and C-type lectins (CTL) to promote either regulatory and/or Th2 responses. For instance, molecules of the soluble egg antigen of *Schistosoma spp.* such as lewis^x^ glycans and lysophosphatitylserine are shown to interact with C type lectin receptors (e.g. DC-SIGN) in cooperation with TLR4 or TLR2, respectively [47], [48], [49], [50]. In addition, the potent immune modulator ES-62 has been shown to interact with TLR4 in a manner distinct from the interaction of TLR4 with LPS [51]. Interestingly, incubation of ES62 with macrophages or dendritic cells *in vitro* can induce low levels of pro-inflammatory cytokines in these cells despite the overall anti-inflammatory effect *in vivo* [4], [10], [51], [52]. As a common theme, these helminth immune modulators stimulate intrinsic cellular signaling pathways like MAPK and Nf-κB that differ from canonical pathways [4], [47], [53]. Future studies need to address whether AvCystatin confers the effects described herein by also interacting with innate receptors.

The intracellular events induced by cystatins are poorly understood. However, it has been shown that parasite-derived cystatins (as well as cystatins of other origin) can alter immune responses by regulation of macrophage functions [12], [13], [54]. We have shown earlier that filarial cystatin alters the cytokine pattern of murine macrophages towards an anti-inflammatory phenotype as compared to cystatins of the free-living nematode *C. elegans* [15]. Chicken cystatin has been reported to modulate macrophage functions *in vitro* and *in vivo*, towards a pro-inflammatory phenotype [55], [56], [57]. Chicken cystatin induced an increased phosphorylation rate of ERK only in IFN-γ primed macrophages, but not in non-primed macrophages [56]. Furthermore, chicken cystatin did not induce p38 phosphorylation. Conversely, the response of mouse macrophages to IFN-γ was shown to be dependent on endogenous cystatin C levels [58]. Macrophages from cystatin C knock out (CysC-/-) mice developed significantly higher levels of IL-10 and lower levels of TNF-α along with reduced NF-κb p65 activation after IFN-γ stimulation. These data suggest a down regulation of IL-10 by endogenous cystatin C. In contrast, recombinant mouse cystatin C added to IFN-γ primed CysC-/- and CysC+/+ macrophages induced significant levels of IL-10, TNF-α and NO. These observations indicate a general immunomodulatory role of cystatins, particularly in IFN-γ primed macrophages. Thus, it is tempting to speculate that the intrinsic capacity of cystatins as immune regulators has been employed and evolutionarily optimized by parasites towards induction of regulatory macrophages, in order to favor their survival in the host.

Intracellular events upon extracellular stimuli are transiently regulated by negative feedback mechanisms. DUSPs play a major role in such a feedback control by dephosphorylating the various MAP kinases [34], [59] and therefore were candidates for the downregulation of AvCystatin-induced kinase activation. The prediction of regulation by phosphatases such as DUSPs by the mathematical model was proven by dedicated experiments. We showed that AvCystatin indeed induced expression of DUSP1, DUSP2 *in vitro* and *in vivo*. Moreover, the mRNA and protein level of DUSP1 and DUSP2 were differentially dependent on p38 and ERK activation. However, the phosphorylation of p38 and ERK as well as the expression of IL-10 was dependent on DUSP1. We therefore assume that DUSP1 is more important than DUSP2 in our system.

Mathematical models are powerful tools for the analysis of regulatory networks. The mathematical approach enabled us to study interconnections between the elements that compose the molecular network. We used perturbation of single components of the system to allow for predictions about the behavior of other components. We present here a model, supported by the data that concentrates mainly on direct feedback mechanisms by phosphatases. Disturbing ERK or p38 in our model revealed that ERK but not p38 directly correlates with the IL-10 production after AvCystatin treatment. This is in line with a recent publication showing a linear correlation between the strength of ERK phosphorylation and IL-10 production in different cell types [60]. In addition, phosphorylation of ERK has a significant role for the strength of IL-10 production in regulatory type 2 macrophages. Therefore, it has been proposed to use ERK as a potential marker for the otherwise highly heterogeneous population of regulatory macrophages [4], [23], [61].

Taken together, our study depicts the intracellular signaling events targeted by the parasite immunomodulator AvCystatin leading to macrophage manipulation. Parasitic nematodes have co-evolved with their host and are therefore well adapted to the host's immune system. Chronic infections with filarial nematodes often last for decades during which worms constantly secrete immunomodulatory cystatins into the surrounding tissue and the blood stream of the infected host [12], [13], [14], [62]. The secretion of cystatins by the worm contributes to immune evasion and it therefore appears that parasites exploit endogenous regulation mechanisms of host cystatins to manipulate macrophage functions.

## Materials and Methods

### Ethics statement

All animal experiments were approved by and conducted in accordance with guidelines of the appropriate committee (Landesamt fuer Gesundheit und Soziales, Berlin, Germany).

### Mice, cell isolation and cultivation

Peritoneal macrophages were isolated from naïve or thioglycollate treated (4d after treatment) 8–12 week old BALB/c mice by standard procedures [63]. *Dusp1* knock out mice were used with permission from Bristol-Myers Squibb, back-crossed for seven generations to C57Bl/6 and bred at the University Hospital Erlangen. Animals were handled in strict accordance with good animal practice as defined by the relevant local welfare bodies, and approved by the authorities of the local government (Berlin, Germany). All experiments were performed in accordance with the German law and guidelines for animal protection. If not otherwise stated cells were cultured in Dulbecco's modified Eagle's Medium (DMEM) supplemented with 10% fetal bovine serum, 1 mM L-Glutamine, and 100 U/ml Penicillin G and 100 µg/ml Streptomycin (cDMEM) at 37°C and 5% CO_2_.

In experiments including inhibitors, these were added to the cell culture 60 min before stimulation. All inhibitors were tested for cytotoxicity in our system by Trypan Blue and propidium iodide exclusion assays and only inhibitor concentrations that ensured viability >85% were used. Following inhibitors were used, PI3K inhibitor (LY294002), p38 inhibitor (SB203580), MEK1/2 inhibitor (U0126), tyrosine kinase inhibitor (genistein), JNK-inhibitor II (all from Calbiochem), actin polymerization inhibitor (cytochalasin D, Sigma). JNK-inhibitor II was tested for functionality in western blot experiments with cell lysates from LPS stimulated macrophages and revealed significant blocking capacity at a concentration of 2 µM (data not shown). In some experiments IL-10 receptor was blocked by applying 20 µg/ml anti-IL-10 receptor antibodies into the culture media (clone 1B1, gift from Dr. Hyun-Dong Chang, Deutsches Rheuma-Forschungszentrum Berlin).

### Protein expression, purification and labeling

AvCystatin and control protein (SNAP) were expressed in *E. coli* and affinity purified under native conditions as earlier described [11]. LPS decontamination was done with Endotrap columns (Profos). Residual LPS concentration was determined by Limulus Amoebocyte Lysate LPS detection kit QCL 1000 (Cambrex). LPS concentrations of protein fractions were below 0.1 EU/ml. To exclude that our AvCystatin effects were due to remaining contaminations in the buffer we used a control made by denaturation of AvCystatin (5 min, 90°C). The ‘denaturated AvCystatin’ control had similar endotoxin values as the functional AvCystatin protein. In some experiments we used (in addition to the denaturated AvCystatin) the purified SNAP-tag protein (New England Biolabs) as a ‘control protein’. The SNAP-tag is a mutant of the DNA repair protein O^6^-alkylguanine-DNA alkyltransferase (hAGT) and has no major side effects on macrophage cultures (own observations). This SNAP-tag protein (20 kDa) has roughly the same size as AvCystatin and was expressed and purified from the same expression system in the same way as AvCystatin. The protein concentrations and endotoxin levels were similar to the AvCystatin protein. AvCystatin was labeled with amine reactive dye DyLight549 and DyLight594 according to the protocol of the manufacturer (ThermoFisher). Degree of labeling revealed ∼1 mole dye per mole AvCystatin.

### Western blot analysis

Cells were seeded in 24 well plates at a cell density of 1.5×10^6^ per well and cultured in DMEM without serum. After 2 h cells were washed to remove unattached cells and remaining macrophages were cultured over night. Cells were again washed twice with fresh medium and stimulated with 0.5 µM recombinant AvCystatin or control treatments. After indicated times cells were treated with lysis buffer (10 mM Tris-HCl pH 7.2, 150 mM NaCl, 1% DOC, 1% Triton X-100, 1 mM PMSF, 50 mM NaF, 1 mM Na-orthovanadat, 50 µg/ml Leupeptin, 4 µg/ml) and 7–30 µg of whole cell extracts were separated by SDS-PAGE. After blotting to nitrocellulose membranes (Whatman), the membranes were blocked for 1 h in TBST (10 mM Tris pH 8.0, 150 mM NaCl, 0.1% Tween 20) with 5% non-fat dry milk and incubated with primary antibodies over night. Membranes were washed and incubated with peroxidase-conjugated secondary antibodies. Signals were detected by chemiluminescence reaction (ECL; Amersham Pharmacia) according to the manufacturer's instructions. Primary antibodies were used against phospho-p44/42 MAPK (Thr202/Tyr204), total-p44/42 MAPK, phospho-p38 MAPK (Thr180/Tyr182), total p38 MAPK, phospho-AKT (Ser473), total AKT, phospho-CREB (Ser133), phospho-STAT3 (Tyr705), phospho-STAT3 (Ser727), phospho-DUSP1 (Ser359), β-actin (all from Cell Signaling Technologies), and DUSP1 (Santa Cruz Biotechnologies).

### ELISA

For Cytokine analysis cells were seeded in 96 well plates at a cell density of 3×10^5^ cells per well and cultured in cDMEM. After 2 h non-attached cells were removed by washing and remaining macrophages were cultured in cDMEM for additional 24 h. After washing cells were stimulated with 0.5 µM AvCystatin or control treatments for 18 h and cell supernatants were analyzed for IL-10 by Enzyme Linked Immunosorbent Assay (ELISA) according to the protocol of the manufacturer (BD Biosciences).

### Real-time PCR

Cells were seeded in 24 well plates at a cell density of 2×10^6^ per well and cultured in cDMEM. After 2 h cells were washed to remove unattached cells and remaining macrophages were cultured over night in cDMEM. Cells were washed with fresh cDMEM and stimulated with 0.5 µM recombinant AvCystatin or control treatments for indicated times. Cell lysis and total RNA isolation was done by applying the innuPREP RNA kit according to suppliers' instructions (AnalyticJena). RNA was reverse transcribed using the high Capacity RNA-to-cDNA kit (Applied Biosystems). The real time PCR reaction was performed using the FastStart Universal SYBR Green Master Mix (Roche) in an ABI 7300 Real-Time PCR System and data were processed with ABI 7300 SDS software (Applied Biosystems). The gene specific primer sequences can be obtained upon request. Ct values of the target genes were normalized to the ct values of the housekeeping gene (PPIA) and expressed as fold induction compared to the untreated control by applying the ΔΔct method.

### Cell staining and flow cytometry

For *ex vivo* analysis, labeled AvCystatin (40 µg/mouse) was injected into the peritoneum of 8-12 week old BALB/c mice. After 20 min PEC were isolated with ice-cold PBS/0,02%BSA and washed 2 times with FACS buffer (PBS, 0,2% BSA, 2 mM EDTA) to eliminate any residual AvCystatin. For *in vitro* analysis, labeled AvCystatin (5 µg/ml) was incubated with 2×10^5^ freshly isolated PEC for 30 min on ice or at 37°C in PBS with 0,02% BSA. Control cells without AvCystatin were treated the same way. After incubation all samples were put on ice and washed 4 times with ice-cold FACS buffer. Cells were stained for 15 min in various combinations with anti-CD4-Pacific blue (clone RM4-5, BD biosciences), anti-CD19-FITC (clone 1D3, eBiosciences), anti-F4/80-APC (clone BM8, eBiosciences), anti-CD11b-FITC (clone M1/70, BD biosciences), anti-CD11c-Pacific blue (clone N418, BD biosciences), anti-Gr1-Bio (clone RB6-8C5, gift from DRFZ, Berlin), streptavidin-PECy7 (BD biosciences). Non-specific binding was prevented by addition of anti-mouse-FcγR (clone 2.4G2, gift from DRFZ). Cells were finally analyzed on a BD FACSCanto flow cytometer (BD biosciences) and results were processed with FlowJo software (Tree Star).

### *In vivo* application and analysis

BALB/c mice were treated i.p. with AvCystatin (20 µg/mouse) or the same amount of denaturated AvCystatin for 1 h, 4 h, or 18 h and PEC were isolated with ice-cold PBS/0,02%BSA. We isolated untouched macrophages from PEC by MACS separation and applied the RNA for expression analysis by quantitative Real-Time PCR. Therefore, PEC were incubated with a cocktail of biotinylated antibodies (anti-CD3 (clone 145-2C11, Miltenyi Biotec), anti-CD19 (clone eBio1D3, eBioscience), anti-Gr1 (clone RB6-8C5, gift from DRFZ, Berlin) and anti-CD11c (clone N418, eBioscience)). Non-specific binding was prevented by addition of anti-mouse-FcγR (clone 2.4G2, gift from DRFZ). After antibody binding cells were washed two times and then incubated with Avidin-coated magnetic beads (Miltenyi) following the manufacturer's protocol. Cell separation was performed with autoMACS (Miltenyi Biotec) and the negative cell fraction (macrophages) used for further analyses. Cell purity of remaining macrophages as determined by CD11b/F4/80 staining and flow cytometry was >85%.

### Confocal microscopy

PEC were isolated with ice-cold PBS/0,02%BSA 20 min after i.p.-injection of DyLight594-labeled AvCystatin. Cells were washed 3 times in FACS buffer, treated with anti-FcγR antibodies and counterstained with anti-CD11b-FITC antibodies. Life cells were directly imaged with a Leica TCS SP2 confocal microscope. Image processing was done with LCS software (Leica) and Adobe Photoshop (Adobe Systems).

### Mathematical model and selection strategy

We adapted and extended our previously published mathematical model of IL-10 production and regulation in macrophages [30] to explain the transient behavior of the MAPK ERK and p38. We hypothesized three underlying regulating mechanisms: 1) IL-10 itself deactivates the MAPKs expression (autocrine feedback). 2) Phosphatases deactivate the MAPK. 3) An independent molecule (i.e., a molecule not activated by AvCystatin) deactivates the MAPK. We conducted a systematic analysis of each of these mechanisms and their possible combinations. A master model illustrates the scope of this analysis (Fig. S2).

This master mathematical model containing regulation reactions of interest was implemented using ordinary differential equations (ODE) in the Systems Biology Markup Language (SBML) format. Within the frame of this master model, 35 more specific models (each representing a specific hypothesis about the underlying feedback mechanism) were generated using the software tool for model generation and discrimination ModelMAGE [64] and tested (Table S1). We fitted each model to the kinetics of IL-10 (mRNA and protein), to IL-10 half-life and to the kinetics of ERK or p38 (for details on the method of fitting, please refer to “Model Fitting”, in this section). We ranked the models using the Aikaike Information Criterium (AIC). We selected the top ranked model and validated this model by comparing its predictions of ERK or p38 kinetics with the respective experimental values. The model included an IL-10 dependent mechanism of transient MAPK activation and IL-10 regulation seen in macrophages after AvCystatin treatment (data not shown). To simplify the mathematical model we assumed that IL-10 could directly act on ERK1/2 and/or p38, whereas in reality a feedback would involve surface-located IL-10 receptors.

In order to test assumption 1, that IL-10 itself regulates the MAPK expression (autocrine feedback), we experimentally blocked the IL-10 receptor by applying anti-IL-10 receptor antibodies and compared the level of IL-10 expression in macrophages after AvCystatin treatment. No effect was seen on IL-10 expression induced by AvCystatin in the absence of IL-10 receptor signaling (data not shown), thus excluding a feedback mechanism by IL-10 itself. In response to this result, we selected the top ranked mathematical model that did not include an IL-10 feedback mechanism (model 15 of Table S1). For details on the method of selection and validation, please refer to “Model selection” and “Model validation”, in this section.

### Model fitting

Fitting was done with COPASI [65]. Each model was fitted to two different data sets. Data set 1 comprised the kinetic data of IL-10 (mRNA and protein) and half-life of IL-10 mRNA, published in Figueiredo 2009, together with p38 (Fig. 3A). Data set 2 comprised the same kinetic data of IL-10 (mRNA and protein) and half-life of IL-10 mRNA, but together with ERK (Fig 3C).

Each model was fitted 8 times to each data set, using different algorithms and parameter ranges. Algorithms for Evolutionary Programming and Simulated Annealing were tested. The free parameters were constrained between 0.0001 and 10 or 0.0001 and 100.

### Model selection

Model discrimination was done with ModelMaGe (http://www.modelmage.org) [64], using the AIC (Aikaike information criterium). AIC measures the goodness of fit of a model and is a tool for model ranking and selection, giving preference to those models that are able to explain the data with a minimum number of free parameters [66].

We calculated the AIC for each model and each fitting method, obtaining 16 different AIC values for each model. We then ranked the models and created a list of the “top 10” best models (according to the AIC) for each fitting method. To select between models, we then computed how often each model occurred among the top 10 lists. We selected the most frequently occurring model and checked its consistency with the experimental data.

We also computed the average of the AIC for each model and created an ordered list containing the 10 models with the lowest AIC average. We compared this list to the previous one and selected the only model that was present in both lists and was consistent with the experimental data. The selected model was model 15. To check the robustness of this result, we also calculated the median of the AIC for each model. The best model consistent with the experimental data was again model 15.

### Model validation

Model validation was done by comparing phospho-p38 kinetics to the corresponding simulation curve for p38, when the model was fitted to data set 1. When the model was fitted to data set 2, the models were validated by comparing phospho-ERK kinetics to the corresponding simulated curve for ERK. The next step was to test a specific feature of the model with a new dedicated experiment (we experimentally tested assumption 1, that IL-10 itself regulates the MAPK expression (autocrine feedback)).

In the specific case of model 15, we checked for other parameter sets that can predict ERK phosphorylation dynamics with a lower difference between the experimental data point and the simulation. We found another parameter set able to fit the model to the data and to predict ERK phosphorylation closer to the experimental data points of phospho-ERK. We then analyzed the sensitivity and robustness of these two parameter sets. We observed that the parameter set that we propose in the manuscript was more robust than the parameter set that gives a closer prediction of ERK. Robustness is believed to be an essential property of biological systems in general [67], [68] and we believe that the particular host-parasite interaction we are studying has been shaped to be robust. The sensitivity analysis showed that the model with a lower error between predicted ERK and experimental values was not able to correctly predict the perturbation experiments. Hence, despite the fact that this parameter set offers an ERK prediction closer to the experimental data, we chose the parameter set that: 1. is more robust; 2. shows a transient trend for phosphorylated ERK; 3. correctly predicts the interconnection between ERK and P38, studied with a sensitivity analysis that is in accordance with the experimental data.

### Statistical analysis

Statistical analysis and graphical output of data was done with the computer program Prism (GraphPad software). The data were first tested with one-way ANOVA test followed by the Bonferroni's multiple comparison test to retrieve statistical significance between test groups.

### Accession number

AvCystatin (EMBL-Bank L43053).

## Supporting Information

Figure S1 AvCystatin stimulated signaling pathways are partially connected but are independently addressed by AvCystatin. Thioglycollate elicited peritoneal macrophages from BALB/c mice were treated with 0.5 µM AvCystatin in the presence of LY294002 (5 µM, PI3K inhibitor), SB203580 (0.5 µM, p38 inhibitor) or U0129 (5 µM, Mek1/2 inhibitor). Cells were pre-incubated with inhibitors for 60 min before addition of AvCystatin. The following controls were also applied: a medium control (medium), a control with denaturated AvCystatin (denat. AvCys) and a control protein (0.5 µM). After indicated times total cell extracts were isolated and applied in western blot analysis using antibodies against phospho-p38, phospho-ERK, phospho-AKT and respective total proteins.
(3.79 MB TIF)

Figure S2 Wiring scheme of the master model of IL-10 production and regulation in macrophages after AvCystatin stimulation. This graphical model represents possible IL-10 regulation mechanisms (regulation of IL-10 through DUSP, IL-10 or an independent molecule (IM)) and comprises the possible mechanism of achieving this regulation. Wiring scheme was performed according to SBGN (http://www.sbgn.org/Main_Page). Black Lines depict the core model, i.e., the fixed reactions present in all model combinations. The grey lines refer to the reactions that vary with the different regulation mechanisms.
(0.38 MB TIF)

Figure S3 Work flow describing the process of model selection. 35 alternative models are generated based on literature and experimental data, fitted to the available experimental data (ERK, p38, and IL-10) and ranked based on the AIC. The best model is selected and checked if it predicts the experimental data. If not, the next best model is selected until having one that fits the data. If yes, a dedicated experiment is done in order to test the specific method of regulation mechanism of the top model. If the model is consistent with this new experiment, then the model will be selected.
(1.69 MB TIF)

Figure S4 Mapping the experimental data from DUSP 1 and DUSP2 with predicted DUSP. The DUSP values of DUSP 1 and DUSP2 from Fig. 8A were converted to arbitrary units and overlay with predicted DUSP from model 15.
(0.32 MB TIF)

Table S1 Mathematical models derived from the master model shown in figure S2. IM: independent molecule, KI: kinase inhibition, PA: phosphatase activation, →: activation, ┤: repression.
(0.05 MB DOC)
