# Supplementary figures and images for: A Helminth Immunomodulator Exploits Host Signaling Events to Regulate Cytokine Production in Macrophages
Source: PLoS Pathog. 2011 Jan 6;7(1):e1001248. doi: 10.1371/journal.ppat.1001248 (PMC3017123; doi:10.1371/journal.ppat.1001248)

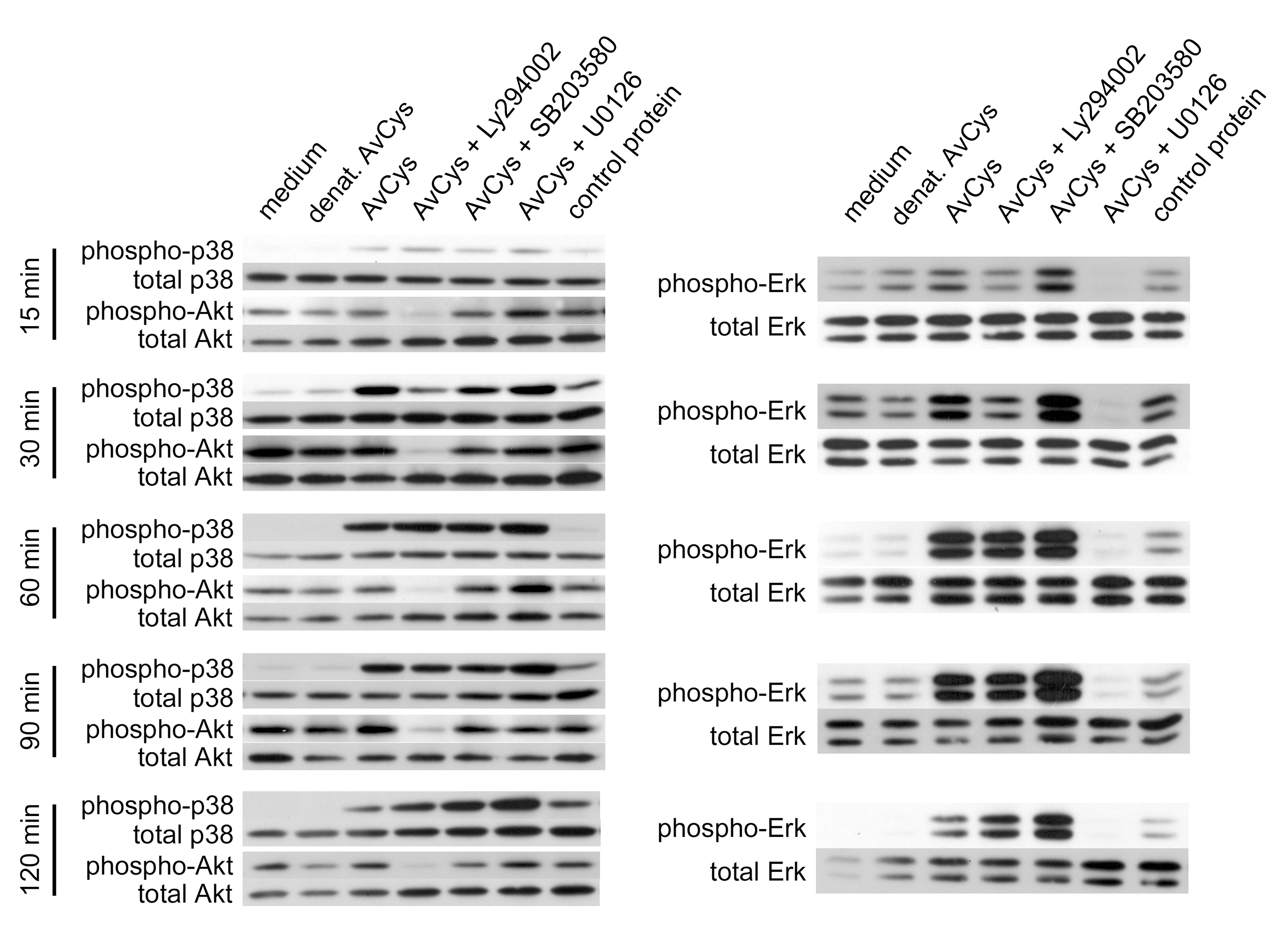

Supplement: Figure S1 — AvCystatin stimulated signaling pathways are partially connected but are independently addressed by AvCystatin. Thioglycollate elicited peritoneal macrophages from BALB/c mice were treated with 0.5 µM AvCystatin in the presence of LY294002 (5 µM, PI3K inhibitor), SB203580 (0.5 µM, p38 inhibitor) or U0129 (5 µM, Mek1/2 inhibitor). Cells were pre-incubated with inhibitors for 60 min before addition of AvCystatin. The following controls were also applied: a medium control (medium), a control with denaturated AvCystatin (denat. AvCys) and a control protein (0.5 µM). After indicated times total cell extracts were isolated and applied in western blot analysis using antibodies against phospho-p38, phospho-ERK, phospho-AKT and respective total proteins. (3.79 MB TIF) [file ppat.1001248.s001.tif]

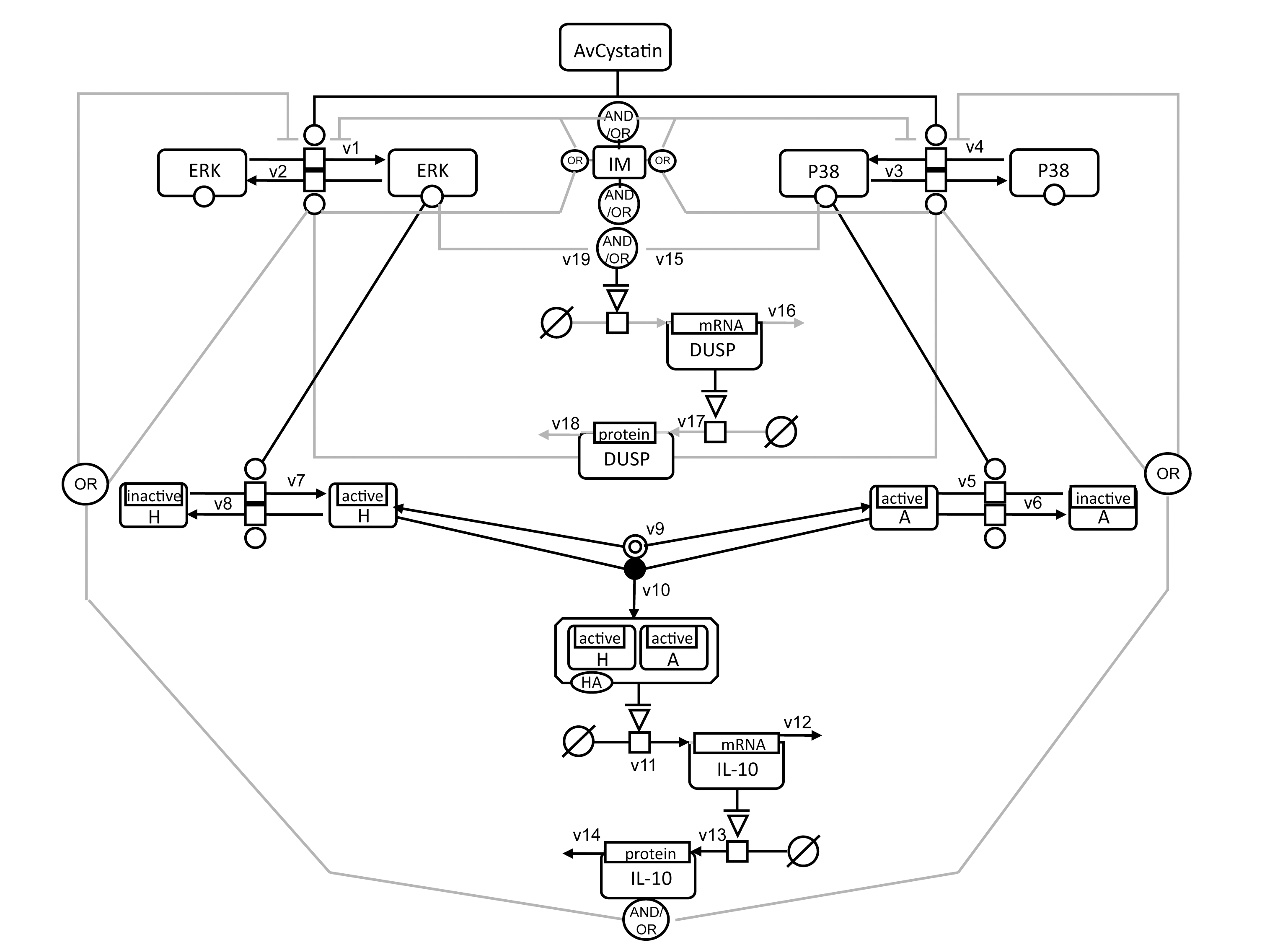

Supplement: Figure S2 — Wiring scheme of the master model of IL-10 production and regulation in macrophages after AvCystatin stimulation. This graphical model represents possible IL-10 regulation mechanisms (regulation of IL-10 through DUSP, IL-10 or an independent molecule (IM)) and comprises the possible mechanism of achieving this regulation. Wiring scheme was performed according to SBGN (http://www.sbgn.org/Main_Page). Black Lines depict the core model, i.e., the fixed reactions present in all model combinations. The grey lines refer to the reactions that vary with the different regulation mechanisms. (0.38 MB TIF) [file ppat.1001248.s002.tif]

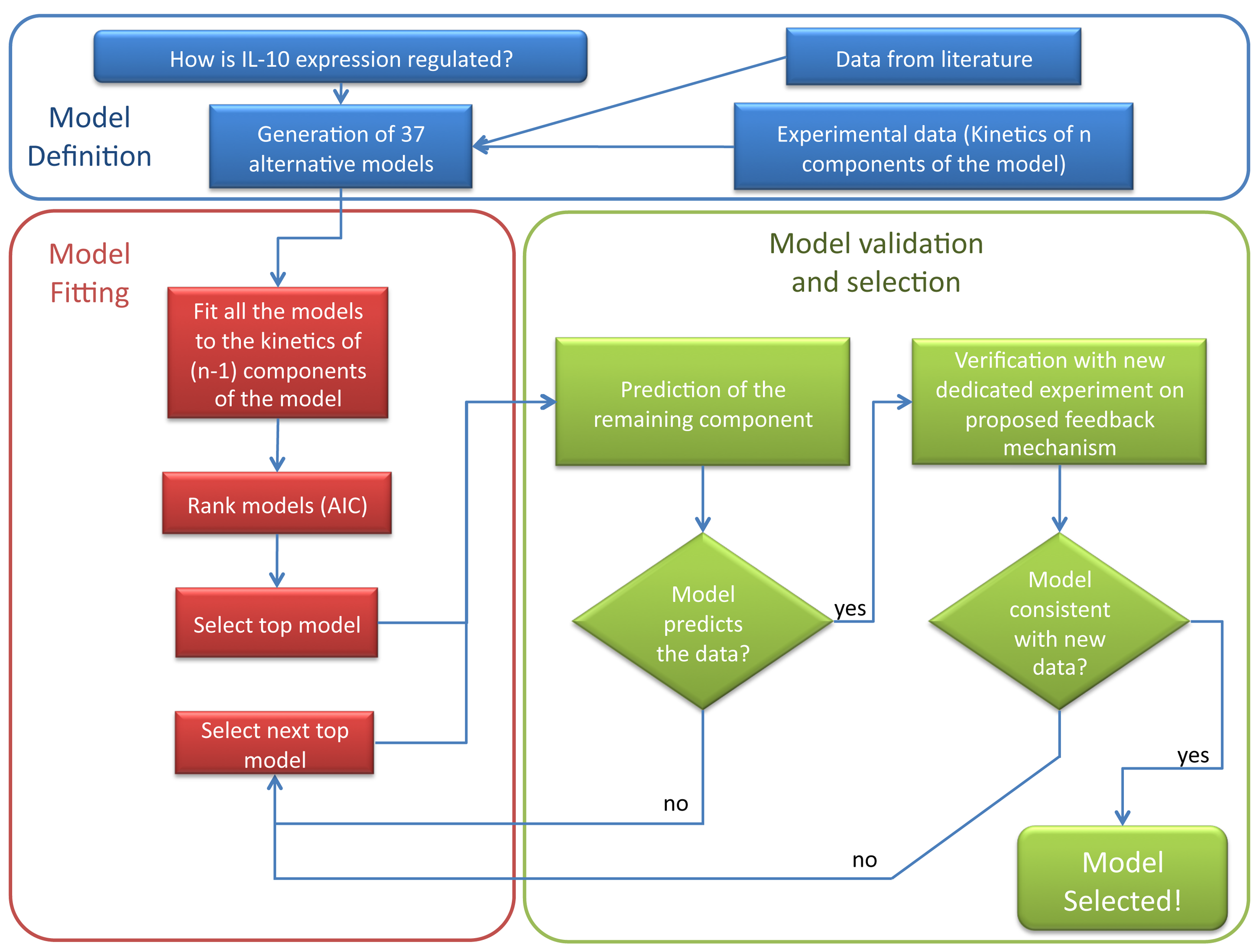

Supplement: Figure S3 — Work flow describing the process of model selection. 35 alternative models are generated based on literature and experimental data, fitted to the available experimental data (ERK, p38, and IL-10) and ranked based on the AIC. The best model is selected and checked if it predicts the experimental data. If not, the next best model is selected until having one that fits the data. If yes, a dedicated experiment is done in order to test the specific method of regulation mechanism of the top model. If the model is consistent with this new experiment, then the model will be selected. (1.69 MB TIF) [file ppat.1001248.s003.tif]

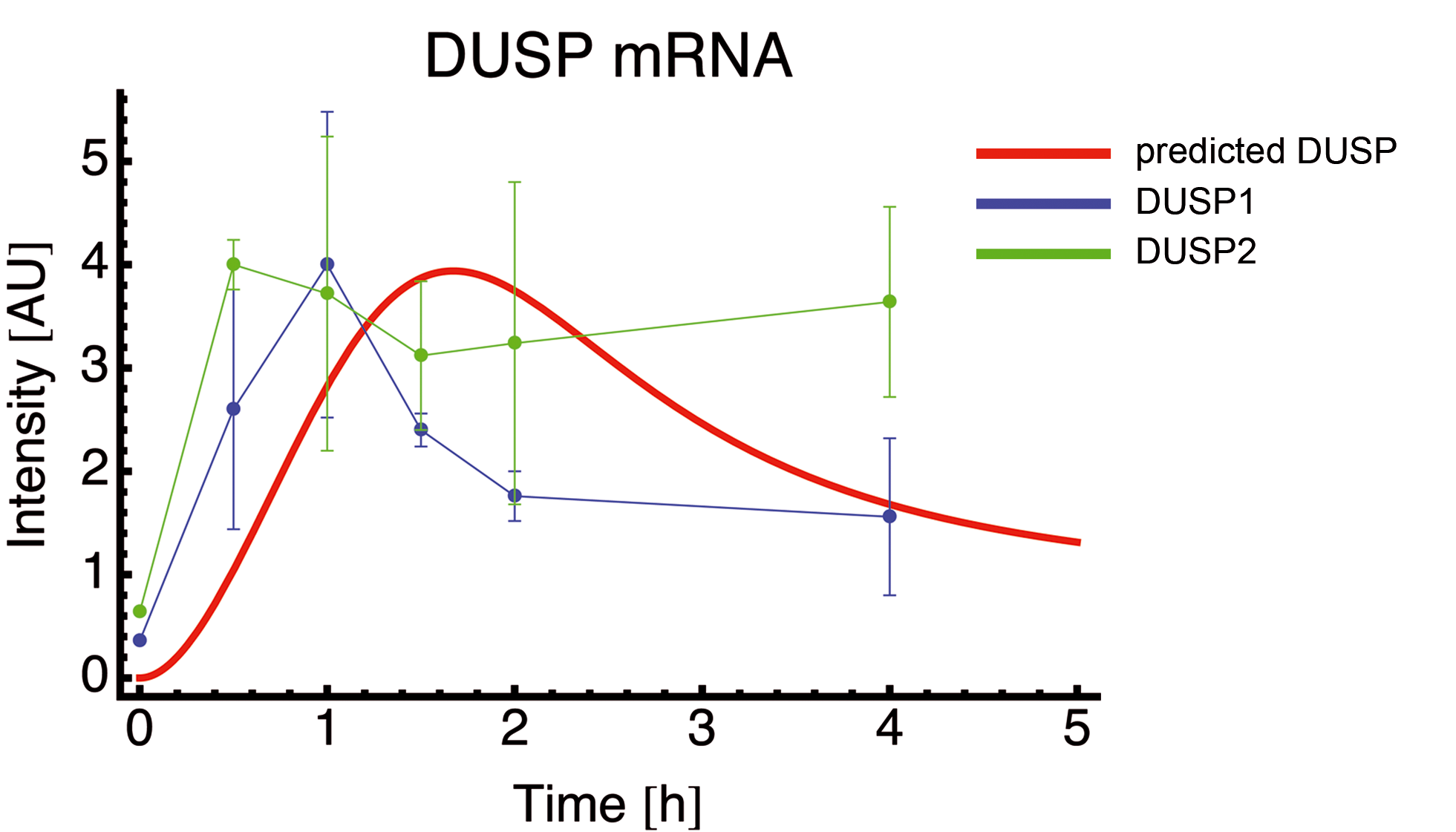

Supplement: Figure S4 — Mapping the experimental data from DUSP 1 and DUSP2 with predicted DUSP. The DUSP values of DUSP 1 and DUSP2 from Fig. 8A were converted to arbitrary units and overlay with predicted DUSP from model 15. (0.32 MB TIF) [file ppat.1001248.s004.tif]
