# Supplementary material for: A Helminth Immunomodulator Exploits Host Signaling Events to Regulate Cytokine Production in Macrophages
Source: PLoS Pathog. 2011 Jan 6;7(1):e1001248. doi: 10.1371/journal.ppat.1001248 (PMC3017123; doi:10.1371/journal.ppat.1001248)
Supplement: Table S1 — Mathematical models derived from the master model shown in figure S2. IM: independent molecule, KI: kinase inhibition, PA: phosphatase activation, →: activation, ┤: repression. (0.05 MB DOC) [file ppat.1001248.s005.doc]

| Model | Model design |
| --- | --- |
| 1 | ERK →DUSP ┤ERK and IL-10 ┤P38 KI |
| 2 | ERK →DUSP ┤ERK and IL-10 ┤P38 PA |
| 3 | ERK →DUSP ┤P38 and IL-10 ┤ERK KI |
| 4 | ERK →DUSP ┤P38 and IL-10 ┤ERK PA |
| 5 | ERK →DUSP ┤(P38 and ERK) |
| 6 | P38 →DUSP ┤P38 and IL-10 ┤ERK KI |
| 7 | P38 →DUSP ┤P38 and IL-10 ┤ERK PA |
| 8 | P38 →DUSP ┤ERK and IL-10 ┤P38 KI |
| 9 | P38 →DUSP ┤ERK and IL-10 ┤P38 PA |
| 10 | P38 →DUSP ┤(P38 and ERK) |
| 11 | (ERK + P38) →DUSP ┤ERK and IL-10 ┤P38 KI |
| 12 | (ERK + P38) →DUSP ┤ERK and IL-10 ┤P38 PA |
| 13 | (ERK + P38) →DUSP ┤P38 and IL-10 ┤ERK KI |
| 14 | (ERK + P38) →DUSP ┤P38 and IL-10 ┤ERK PA |
| 15 | (ERK + P38) →DUSP ┤(P38 + ERK) |
| 16 | IL-10 ┤(ERK and P38) KI |
| 17 | IL-10 ┤(ERK and P38) PA |
| 18 | IM ┤ERK KI and IL-10 ┤P38 KI |
| 19 | IM ┤ERK PA and IL-10 ┤P38 KI |
| 20 | IM ┤ERK KI and IL-10 ┤P38 PA |
| 21 | IM ┤ERK PA and IL-10 ┤P38 PA |
| 22 | IM ┤ERK KI and P38 →DUSP ┤P38 |
| 23 | IM ┤ERK PA and P38 →DUSP ┤P38 |
| 24 | IM ┤ERK KI and ERK →DUSP ┤P38 |
| 25 | IM ┤ERK PA and ERK →DUSP ┤P38 |
| 26 | IM ┤P38 KI and IL-10 ┤ERK KI |
| 27 | IM ┤P38 PA and IL-10 ┤ERK KI |
| 28 | IM ┤P38 KI and IL-10 ┤ERK PA |
| 29 | IM ┤P38 PA and IL-10 ┤ERK PA |
| 30 | IM ┤P38 KI and ERK →DUSP ┤ERK |
| 31 | IM ┤P38 PA and ERK →DUSP ┤ERK |
| 32 | IM ┤P38 KI and P38 →DUSP ┤ERK |
| 33 | IM ┤P38 PA and P38 →DUSP ┤ERK |
| 34 | IM ┤(P38 and ERK) KI |
| 35 | IM ┤(P38 and ERK) PA |
